# Accelerometer‐based heart rate adjustment for ambulatory stress research

**DOI:** 10.1111/psyp.14721

**Published:** 2024-11-19

**Authors:** Sjors R. B. van de Ven, Martin J. Gevonden, Matthijs L. Noordzij, Eco J. C. de Geus

**Affiliations:** ^1^ Department of Biological Psychology Vrije Universiteit Amsterdam The Netherlands; ^2^ Department of Psychology, Health and Technology University of Twente Enschede The Netherlands

**Keywords:** accelerometer, additional heart rate, ambulatory assessment, autonomic nervous system, heart rate, stress

## Abstract

Using heart rate (HR) measurements to detect mental stress in naturalistic settings is hampered by the physiological impact of hemodynamic and metabolic demands. Correcting HR for these demands can help isolate fluctuations in HR associated with psychosocial stress responses, a concept termed additional heart rate (aHR). This study examined whether adding predictors for posture, activity type, and lagged movement intensity for the prolonged impact of physical activity (PA) improved aHR estimation across various manipulations of mental stress, posture, and PA in a controlled laboratory environment (*n* = 197). Accelerometer signals were used to obtain the movement intensity and to classify posture and activity type. Posture, activity type, and lagged movement intensity each led to a significant improvement in HR estimation, as measured by adjusted *R* ^2^ and root mean squared error. However, HR was overestimated during quiet sitting. The aHR, computed as the difference between observed and predicted HR, generally underestimated observed task‐baseline reactivity but was sensitive to individual differences in reactivity to mental stressors. Between‐subject correlations of aHR with task‐baseline reactivity ranged from 0.62 to 0.93 across conditions. On a within‐subject level, the ability of aHR to differentiate between exposure to physical stress and mental stress was limited (recall = 0.32, precision = 0.31), but better than that of observed HR (recall = 0.02, precision = 0.02). Future research should explore the potential of this novel aHR estimation method in differentiating physical and mental demands on HR in daily life, and its predictive value for health outcomes.

## Abstract 2

Conventional accelerometer‐based additional heart rate focuses solely on concurrent movement intensity to predict heart rate. This study introduces a novel method to correct heart rate not only for ongoing physical activity but also for postural changes, type of physical activity, and past physical activity. This approach enhances the ability to differentiate between the impact of physical and mental demands on heart rate in daily life.

## 1 INTRODUCTION

Exposure to psychosocial stressors prompts cardiovascular responses governed by the autonomic nervous system, such as an increase in heart rate (HR) and blood pressure. Prolonged or recurrent activation of these responses can adversely affect physiological functioning (Epel et al., 2018). The pathophysiological effects of chronic stress are considered to be a common risk factor for various diseases, including cardiovascular diseases (Cohen et al., 2007; Kivimäki & Steptoe, 2018; Rosengren et al., 2004). Identification of reliable biomarkers for maladaptive physiological stress responses is crucial for advancing our understanding of the complex interplay between stressors and long‐term health outcomes. HR, the number of myocardial contractions per time unit, stands out as a compelling candidate due to its sensitivity to modulation by both the sympathetic and parasympathetic branches of the autonomic nervous system. However, HR measurements in naturalistic settings are strongly confounded by the ubiquitous influence of physical activity (Grossman et al., 2004). Studies involving HR measurements in ambulatory settings therefore tend to exclude all data when participants are physically active (e.g., Healey et al., 2010; Houtveen & de Geus, 2009; Pieper et al., 2007; Vrijkotte et al., 2000). However, stress exposure may impact how much someone engages in physical activity (Schultchen et al., 2019; Stults‐Kolehmainen & Sinha, 2014), and the reverse is also true (Burg et al., 2017). Some people tend to engage less in physical activity in response to experienced stress, whereas other people use physical activity as an outlet to relieve experienced stress. As a consequence, removing all data during physical activity may lead to a loss of relevant data in the context of physiological stress responses and recovery. This highlights the critical need to unravel the effects of physical activity on HR, rather than excluding all data during physical activity.

To meet the increased need for oxygen (or “metabolic demand”) during physical activity, the autonomic nervous system induces an increase in cardiac output (CO), the product of stroke volume and HR (Nystoriak & Bhatnagar, 2018; Shepherd, 1987). HR is known to increase linearly with metabolic demand, regardless of sex and age (Schrack et al., 2014), as opposed to stroke volume which can show several patterns in response to increasing metabolic demand but often plateaus at 40%–50% VO_2_max (Vella & Robergs, 2005). The sinoatrial (SA) node generates an intrinsic HR that usually ranges from 100 to 110 contractions per minute (Klabunde, 2011), but the actual firing rate of the SA node is influenced by opposing effects of the two branches of the autonomic nervous system. HR during rest, typically between 55 and 85 contractions per minute, is primarily influenced by the vagal nerve of the parasympathetic branch. Cardiac vagal nerve activity lowers the excitability of the SA node by releasing acetylcholine, a process that leads to a reduction in HR. When metabolic demand increases, however, chronotropic cardiac vagal nerve activity decreases, leading to an increase in HR, and hence CO. In addition, an increase in metabolic demand triggers the activation of the sympathetic branch. Activation of the sympathetic nervous system increases the firing rate of the SA node, as well as myocardial contractility and stroke volume. These cardiac effects, called chronotropic and inotropic effects, respectively, further contribute to the increase in CO in response to physical activity to meet the increased tissue need for oxygen.

### 1.1 Additional heart rate and its estimation methods

As metabolic demand is determined by physical workload, physical workload is a strong predictor of changes in HR, with within‐subject correlations of physical workload and HR ranging from 0.70 to 0.81 (Myrtek et al., 1996, 2005). However, the residual variation in HR also carries significant information. In Blix et al. (1974) coined the term “additional heart rate” (aHR) to describe the phenomenon of HR that exceeds the expected HR based on the physical workload. The HR in exaggeration of this expected HR was then attributed to psychological effects on the autonomic nervous system. Psychological effects can reflect mental effort as well as emotional arousal and are often summarized under the term “mental stress.” aHR conceptually relies on the assumptions that the effect of a combination of physical workload and mental stress corresponds to the linear sum of the effects evoked by each of them (Myrtek & Spital, 1986; Roth et al., 1990) and that the physical workload component can be reliably estimated (Ebner‐Priemer et al., 2007). If these assumptions are met, discrepancies between the estimated workload‐related fluctuations in HR and the observed HR (oHR) can be used for the quantification of mental stress, even during physical activity. This would be highly beneficial for recordings in naturalistic settings where physical activity is not under experimental control. The idea that aHR could reflect mental stress was confirmed by associations of aHR with perceived stress (Brown et al., 2018; Linssen et al., 2022; Loeffler et al., 2016), perceived tension (Verkuil et al., 2016), perceived intensity of worries (Verkuil et al., 2016) and salivary cortisol concentrations (Loeffler et al., 2016). However, despite early suggestions for the need for appropriate separation of the physiological effects of physical and mental stressors (Gliner et al., 1979; Langer et al., 1985; Myrtek et al., 1996; Payne & Rick, 1986; Sherwood et al., 1986; Turner et al., 1983, 1988; Turner & Carroll, 1985), the implementation of aHR in naturalistic settings has been scant.

Detection of aHR periods is performed by first estimating the impact of physical workload on HR and then subtracting the workload‐related HR from the oHR. Various approaches have been proposed to model the impact of physical workload on HR in the context of aHR (for detailed overview, see Brouwer et al., 2018). First, oxygen consumption was introduced as a measure of physical workload (e.g., Blix et al., 1974; Carroll et al., 1986; Gliner et al., 1979; Turner & Carroll, 1985) because oxygen consumption is known to be linear with HR across the normal physiological range. However, the application of oxygen consumption is limited outside of artificial laboratory settings as it involves breathing through a mask. Minute ventilation through inductive plethysmography was proposed as a less invasive alternative to oxygen consumption (Wilhelm & Roth, 1998) based on the strong agreement between the two (Delistraty et al., 1991). More recently, motion sensors that capture the acceleration of bodily movement along multiple axes (henceforth referred to as accelerometers) have been applied as a measure of physical workload (Brown et al., 2018; Ebner‐Priemer et al., 2007; Kusserow et al., 2013; Linssen et al., 2022; Myrtek et al., 2005; Prill & Fahrenberg, 2007; Verkuil et al., 2016; Yang et al., 2017). Given the small size and non‐invasiveness of accelerometers, this provides the least burdensome option for monitoring physical workload in naturalistic settings.

### 1.2 Challenges in accelerometer‐based aHR

The primary challenge in estimating workload‐related HR using accelerometers is that acceleration of the body in its three major axes does not directly map to physical workload (Brouwer et al., 2018). Summary scores like the vector magnitude (VM) of three accelerometer axes (Qasem et al., 2012) quantify the overall movement intensity. However, a given movement intensity measured during cycling and during walking may not directly correspond to the same physical workload. The distinct relationships between movement intensity and physical workload for various activity types make it difficult to directly estimate HR based on overall movement intensity (Bonomi et al., 2009). Furthermore, posture changes have a large hemodynamic impact (Baldi et al., 2007; Edgell et al., 2012; Patel et al., 2016; Rossberg & Peňaz, 1988) but are not accompanied by a large change in movement intensity (Kusserow et al., 2013). The physiological effects of posture changes are therefore susceptible to being misclassified as physiological effects of mental stress when not taking posture into account. Methods that incorporate posture and type of physical activity when estimating physical workload have been found to outperform methods that only use overall movement intensity (Altini et al., 2015). Consequently, as suggested by Brouwer et al. (2018), a promising approach to estimate the impact of physical activity on HR would be to first utilize accelerometer signals for classification of postures and activity types and then use the accelerometer signals to predict the HR corrected for these different postures and activity types.

The second challenge in estimating physical workload‐related HR using accelerometers is the prolonged physiological impact of physical activity. The effects of physical activity on the cardiovascular system, particularly when the level of activity is moderate to vigorous, do not stop at the exact moment of cessation of activities. Post‐exercise effects on blood pressure (Cornelissen et al., 2010; Halliwill et al., 2013; Kenney & Seals, 1993) and HR (Halliwill et al., 1996; Peçanha et al., 2017; Seiler et al., 2007) may be present that can continue for minutes (or even hours) after activity has ceased (Romero et al., 2017). Estimation of HR based on concurrent movement intensity extracted from accelerometers fails to capture this prolonged impact of physical activity. Consequently, physical activity with sufficient intensity to induce a prolonged HR increase leads to an underestimation of HR if the estimate is only based on concurrent movement intensity (Yang et al., 2017). This demonstrates the necessity to explicitly model the prolonged physiological impact of physical activity in the context of aHR.

### 1.3 Application of aHR

A primary application of aHR is to differentiate cardiovascular responses caused by psychosocial factors from functional cardiovascular responses (to meet metabolic demands or to counteract posture‐induced changes in gravitational pull on the blood). This is a critical mission for ambulatory stress physiology because nearly all models of stress‐related disease, including the modern allostatic load variants (Epel et al., 2018), go back to the idea that prolonged anticipatory responses to expected perturbations (allostasis) that do not actually occur can lead to counterregulatory processes that affect disease risk factors (McEwen, 2006). aHR as a disease risk factor is derived from Obrist's (1976) early conceptualization of allostasis gone haywire as “tissue overperfusion” (Langer et al., 1985; Obrist, 1976). According to Obrist's (1976) concept, the cardiovascular system responds to stressful situations in a metabolically unjustified manner (Obrist, 1976), namely the heart pumping blood in excess of what the tissues actually need. Tissue overperfusion was hypothesized to contribute to the etiology of cardiovascular diseases through counterregulatory processes involving structural changes in the vascular anatomy (Obrist, 1976). aHR therefore reflects “unjustified” cardiovascular responses that could contribute to allostatic load and future disease, as opposed to functional cardiovascular responses that meet true changes in metabolic demand or maintain blood pressure following postural change. Detection of the frequency and duration of aHR during prolonged recordings in daily life situations could help explain why some individuals are at higher risk for stress‐related disease.

The rare applications of aHR in previous studies have mostly used a between‐subject approach (e.g., Blix et al., 1974; Ebner‐Priemer et al., 2007; Pfaltz et al., 2015; Prill & Fahrenberg, 2007; Sims & Carroll, 1990; Wilhelm & Roth, 1998). Between‐subject applications, for example, explore whether individuals with panic disorder are characterized by more aHR than healthy controls (Pfaltz et al., 2015) or whether individuals with bipolar disorder are characterized by higher levels of aHR in comparison with healthy controls (Ebner‐Priemer et al., 2007). We believe this between‐subject application is still viable in future research, especially in longitudinal studies that determine whether aHR can predict future health outcomes. However, with the increased use of ambulatory assessment in psychophysiology, within‐subject applications also have started to appear (Brown et al., 2018; Kusserow et al., 2013; Linssen et al., 2022; Myrtek et al., 2005; Verkuil et al., 2016). These within‐subject applications thus far have mainly aimed to investigate the concurrent correlates of aHR such as experienced stress (Brown et al., 2018; Linssen et al., 2022), affective state (Myrtek et al., 2005) or contextual factors (Yang et al., 2017). The main within‐subject use case we envision would be to provide triggers based on the duration and magnitude of aHR, including biologically triggered experience sampling as suggested by Hoemann et al. (2023) or just‐in‐time adaptive interventions as suggested by Schwerdtfeger and Rominger (2021). Biologically triggered experience sampling could aid in exploring the within‐subject relationships between subjective experiences and physiological responses by including an assessment of prevailing affective state (Hoemann et al., 2020).

### 1.4 The present study

In summary, previous research has confirmed the potential of aHR for the detection of mental stress, but there remain substantial methodological shortcomings in the estimation of aHR. Models estimating HR based on accelerometers could improve their accuracy by taking posture, type of physical activity, and concurrent as well as recent movement intensity into account (Altini et al., 2015; Bonomi et al., 2009; Kusserow et al., 2013; Yang et al., 2017). Hence, this study aims to advance the estimation of aHR by means of a three‐step procedure. First, we use a machine learning algorithm to classify posture (sitting, standing, or lying) and activity type (walking, jogging, cycling, or walking stairs) from triaxial accelerometer signals. Second, we predict HR on an individual level using concurrent movement intensity, posture/activity type class, and past movement intensities. We denote this predicted HR (pHR) as “movement‐related heart rate,” as an umbrella term that encompasses the (prolonged) effect of physical activity and postural change on HR. Third, we subtract the movement‐related HR from the actual oHR to obtain the aHR.

We validate the aHR against a ground‐truth measure for physiological reactivity in laboratory setting (task‐baseline reactivity) by examining the between‐subject correlation between aHR and task‐baseline reactivity to mental and physical stressors. We further test the within‐subject ability of aHR to differentiate physical stress from mental stress in situations where the ground truth of stress exposure is not known. We compared this to the ability of the observed (uncorrected) HR to do the same. The overarching expectation is that correction for concurrent and recent movement intensity and posture/activity type classes will significantly improve the estimation of movement‐related HR fluctuations and therefore of aHR. The use of the improved aHR will increase our ability to differentiate between the effects of physical and mental demands on HR, which could greatly assist us in non‐invasive monitoring of occurrence of mental stress in naturalistic settings.

## 2 METHODS

### 2.1 Study population

This study combined two data sets collected at the Vrije Universiteit Amsterdam between 2011 and 2022. The first study included 72 undergraduate students between 18 and 27 years old, and the second study included 128 participants between 18 and 48 years old. Exclusion criteria for both studies included BMI higher than 30, cardiac diseases, thyroid diseases, high blood pressure, high cholesterol, or use of medication that is known to affect the autonomic nervous system (e.g., beta‐blockers, antidepressants, or anticholinergics). To account for hormonal changes, all female participants participated within 2 weeks after the last day of their menstrual cycle. All participants gave written informed consent to participate and the study protocols were submitted to and approved by the VUmc Medical Ethical Committee (METc VUmc #2017.374).

### 2.2 Materials

In both studies, the VU‐AMS5fs device (Vrije Universiteit, Amsterdam, the Netherlands) was used to monitor the activity of the autonomic nervous system (de Geus et al., 1995; de Geus & Gevonden, 2023). This device simultaneously records the electrocardiogram (ECG) and impedance cardiogram (ICG) through five pregelled Ag/AgCl electrodes with a sampling frequency of 1000 Hz. The skin was pretreated with alcohol to improve electrode adhesion and minimize resistance. One electrode that contributes to both ECG and ICG was placed on the sternum between the sternal ends of the collarbones. A second ECG electrode was positioned near the apex of the heart over the ninth rib on the left lateral chest. A second ICG electrode was placed slightly below the xiphoid process of the sternum. Two ICG current electrodes were placed on the vertebral column on the back. The first ICG current electrode was placed on the cervical vertebra C4, located at least 3 cm above the combined ECG/ICG electrode at the sternum. The second ICG current electrode was placed between the thorax vertebrae T8 and T9, at least 3 cm below the ICG electrode at the xiphoid process.

The VU‐AMS5fs device comes equipped with an ADXL330 triaxial accelerometer (One Technology Way, Norwood, MA, USA) that measures acceleration along the anterior–posterior (*x* axis), vertical (*y* axis), and mediolateral (*z* axis) planes. The triaxial accelerometer has a minimum full‐scale range of ±3 g and sensitivity of 300 mv/g and minimized cross‐axis sensitivity. The device was worn in a dedicated, tight‐fitting carrying bag held at the left hip by means of a belt.

### 2.3 Procedure

Both studies adopted a similar study procedure. Given the aforementioned agreement in terms of materials, the data from both studies could be merged. Both study protocols lasted approximately 2–2.5 h. In the first study, the start time of the laboratory session was standardized relative to the waking time of the participants. Specifically, the session was scheduled within the first 2 h after awakening. The second study did not impose any restrictions on the starting time of the laboratory sessions which varied between 10:00 a.m. and 4:00 p.m.

The goal of the laboratory session was to impose artificial variation in posture, variation in type and intensity of physical activity, and variation in levels of mental stress. An overview of the conditions and the order of the conditions for both studies can be found in Table 1. Furthermore, a detailed explanation of the procedure of the second study can be found in van der Mee et al. (2021).

**TABLE 1:** Order and duration of all experimental conditions for both studies.

Condition	Duration	Activity class	Mental stress
Study 1			
Baseline	4 min	Sitting	‐
Standing	3 min	Standing	‐
Lying down	3 min	Lying down	‐
Sitting	3 min	Sitting	‐
Lying down	3 min	Lying down	‐
Standing	3 min	Standing	‐
Sitting	3 min	Sitting	‐
Tone Avoidance	3 min	Sitting	Yes
Walking outside at leisurely pace	2 min	Walking	‐
Walking outside while talking	2 min	Walking	‐
Walking stairs	4 min	Stairs	‐
Recovery (sitting)	3 min	Sitting	‐
Ergometer 50 Watt with 60 RPM	4 min	Cycling	‐
Ergometer 100 Watt with 60 RPM	4 min	Cycling	‐
Ergometer 150 Watt with 60 RPM	4 min	Cycling	‐
Recovery (sitting)	3 min	Sitting	‐
Treadmill (4.5–5 km/h)	4 min	Walking	‐
Treadmill (6–6.5 km/h)	4 min	Walking	‐
Treadmill (7.5–8 km/h)	4 min	Jogging	‐
(Recovery (sitting))	3 min	Sitting	‐
(Vacuum cleaning)	3 min	Walking	‐
(Moving plates)	3 min	Standing	‐
Study 2			
Lying down	3 min	Lying down	‐
Standing	3 min	Standing	‐
Sitting	3 min	Sitting	‐
Tone Avoidance	4 min	Sitting	Yes
Recovery (sitting)	2 min	Sitting	‐
Short sing‐a‐Song Stress Test	6.5 min	Sitting	Yes
Recovery (sitting)	2 min	Sitting	‐
Paced Auditory Serial Addition Test	4 min	Sitting	Yes
Recovery (sitting)	2 min	Sitting	‐
Raven's progressive matrices	4 min	Sitting	Yes
Walking at natural pace	2 min	Walking	‐
Fast walking	2 min	Walking	‐
Cycling	4 min	Cycling	‐
Walking stairs	4 min	Stairs	‐
Recovery (standing)	2 min	Standing	‐
Dish washing	2 min	Standing	‐
Vacuum cleaning	2 min	Walking	‐
Recovery (sitting)	2 min	Sitting	‐
Tone Avoidance	4 min	Sitting	Yes
Recovery (sitting)	2 min	Sitting	‐
Paced Auditory Serial Addition Test	4 min	Sitting	Yes
Treadmill (4.5–5 km/h)	4 min	Walking	‐
Treadmill (6–6.5 km/h)	4 min	Walking	‐
Treadmill (7.5–8 km/h)	4 min	Jogging	‐
Treadmill (3.7–4 km/h)	3 min	Walking	‐
Recovery (sitting)	3 min	Sitting	‐

### 2.4 Data processing

HR was obtained from the ECG using an automated R wave peak detector in the VU‐AMS5fs software (VU‐DAMS for Windows, version 5.3.1, Vrije Universiteit, Amsterdam, Netherlands). Automated scoring of the R waves was double checked by visual inspection of the ECG signal. Deviant beats (too short or too long considering the local average and variance) were corrected by interpolation or excluded by marking these beats as artifacts. The average HR was obtained from the RR interval time series over 30 s windows, analogous to previous studies estimating the HR from accelerometer signals (Brown et al., 2018; Verkuil et al., 2016). In addition, 30 s is considered to be a reasonable compromise between capturing a meaningful variation in physiological parameters and providing a stable estimate during both rest and physical activity.

**FIGURE 1:**
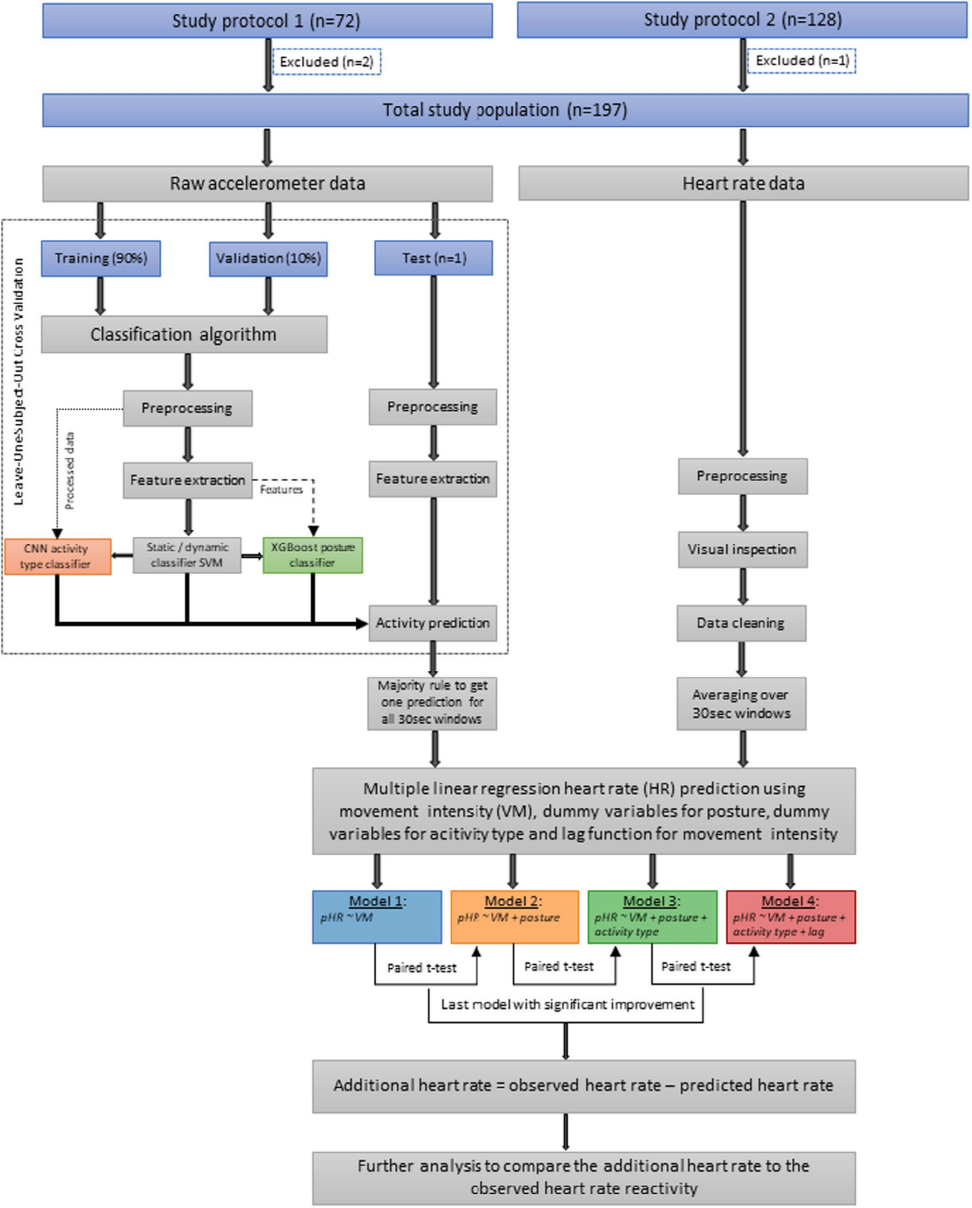
Flowchart of the data processing pipeline showcasing the inclusion of participants, the algorithm for posture and activity type recognition, the processing steps for the heart rate data, the statistical testing of the four different heart rate prediction models, and the further analysis with the best model.

The triaxial accelerometer signals were filtered with a fourth‐order 5‐Hz low‐pass Butterworth filter and subsequently downsampled from 1000 to 100 Hz with an infinite impulse response (IIR) antialiasing filter. The accelerometer signals were only corrected for gravity in order to compute the VM signal. Gravity correction was performed by applying a fourth‐order 0.1‐Hz high‐pass Butterworth filter to a copy of all three acceleration signals. Subsequently, the same filtering and downsampling steps were applied to the high‐pass filtered signals. The VM was then calculated as the root mean square of the preprocessed and gravity‐corrected accelerometer signals. The individual preprocessed accelerometer signals, as well as the VM, were cut into 6 s epochs overlapping by 50%. After feature extraction and classification (see Section 2.5), the conversion from 10 predicted epochs per 30 s to one prediction per 30 s window was performed by means of the majority rule. The category that was predicted the most frequently for these 30 s was chosen as the type of activity for this window. See Figure 1 for a flowchart of the data processing pipeline.

### 2.5 Activity recognition

As mentioned in the introduction, a promising approach to estimate the impact of physical activity on HR would be to first classify posture and activity type based on accelerometer signals and then predict the HR corrected for these different activity classes (Altini et al., 2015; Brouwer et al., 2018). The approach in this study was based on Altini et al. (2015), where accelerometer signals were first divided into either “sedentary” or “active” activities. Subsequently, sedentary activities were subdivided into postures, and active activities were subdivided into activity types. In this study, an algorithm inspired by this two‐level classification concept was created. Specifically, we created a tailored version of the two‐level classification algorithm by Coelho et al. (2022). The algorithm was tailored to the range of postures (sitting, standing, and lying) and activity types (walking, jogging, cycling, and walking stairs) observed in the study protocols. The way in which the experimental conditions were grouped in these activity classes can be found in Table 1.

A leave‐one‐subject‐out cross‐validation (LOSO‐CV) framework was adopted to create an individual activity recognition algorithm for each participant. All data from one participant were held out, while all data from the rest of the participants (equaling *n* = 196) were included as training or validation data. The data of the rest of the participants were divided into a training set and a validation set by means of a 90%/10% split across participants. This way, unseen data from new participants could be used to avoid overfitting on comparable data points of the same condition within one participant.

For each axis, the following features were calculated: mean, maximum, minimum, range, variance, standard deviation, skewness, kurtosis, inclination, and zero‐crossing rate. Additionally, the mean, variance, and standard deviation were calculated for the VM as well as the pairwise correlation between the three axes (e.g., correlation of *x* axis and *y* axis). The features were used to separate all 6 s epochs in either static or dynamic activities using a linear support vector machine (SVM) using Scikit‐learn (version 1.2.2, Pedregosa et al., 2011) in Python. The optimal value of the cost hyperparameter (ranging between 2 and 16) was determined with a fivefold cross‐validation. After the separation of static and dynamic activities, static and dynamic activities were classified separately.

Static activities were classified into three posture classes (i.e., sitting, standing, and lying) using a gradient‐boosting decision tree algorithm XGBoost (version 1.7.4, Chen & Guestrin, 2016). Hyperparameters were selected using a Bayesian optimization algorithm (version 0.2.7, Bergstra et al., 2013). The validation set of unseen data was included during the hyperparameter tuning phase to avoid overfitting. The following search space was defined for the hyperparameters: max_depth (2–6), gamma (0.5–10), eta (0.05–0.3), reg_alpha (1–50), reg_lambda (1–10), subsample (0.8–1), colsample_bytree (0.5–1) and min_child_weight (0–10). The performance metric used for selection of the best hyperparameters was the weighted F1 and the maximum number of evaluations was set at 10 times the number of ordinal hyperparameters, totaling 80 evaluations.

Dynamic activities were classified into four activity type classes (i.e., walking, jogging, cycling, and walking stairs) using a convolutional neural network (CNN) in Keras (version 2.11.0, Chollet & Others, 2015). Analogous to Coelho et al. (2022), the CNN model was trained using a stochastic gradient descent optimizer with predetermined hyperparameters. The learning rate was set to 0.01, the learning rate decay was set to 1e‐4, and the Nesterov momentum was set to 0.9. The CNN training phase was limited to 100 iterations.

The results of the activity recognition algorithm were calculated for each participant individually given the LOSO‐CV framework. All performance metrics were reported as average ± standard deviation. The performance metrics included were accuracy, weighted F1, weighted precision, and weighted recall. We opted for weighted averages for F1, precision, and recall, as there was variability between participants for the availability of data for each of the seven classes. Not all participants performed all experimental conditions, therefore, these performance metrics could not be calculated for each activity class for each participant.

### 2.6 HR prediction

Four distinct regression models were created per participant to estimate movement‐related HR. These regression models incorporated four separate predictor groups subsequently: overall movement intensity, posture class, activity type class, and average movement intensity in preceding 30 s windows. HR prediction was performed for each participant individually by means of a fivefold cross‐validation approach. Eighty per cent of the data of a participant were randomly selected for model creation, while the remaining 20% of a participant were held out for prediction purposes. This is repeated five times to obtain a continuous prediction. This approach avoids “double dip,” where the same data are used for both model training and HR prediction. Furthermore, the implementation of a hold‐out set for predicting minimizes the risk of reporting on artificial improvements due to overfitting on additional predictors. All models were created using the ordinary least squares function from Python's “statsmodel” package.

In the first model, the overall movement intensity, as measured by the VM, was used exclusively to estimate the HR (see Model 1). In this equation, *pHR* stands for the pHR and *VM* stands for the observed VM. $\beta_{0}$ is the intercept term that represents the expected HR in the absence of movement. $\beta_{1}$ is the slope coefficient that represents the change in HR for a unit increase in the VM (in g) over the entire data set. *ε* is the error term that captures the random variation in HR that cannot be explained by the VM. The subscript i represents all datapoints and ranges from 1 to the number of datapoints.

Model 1 (pHR ~ VM):

$${\mathrm{pHR}}_{i}=\beta_{0}+\beta_{1}\;{\mathrm{VM}}_{i}+\varepsilon_{i}.$$

In the second model, posture classes were included as dummy variables. The posture classes were included for all 30 s windows, including the windows when participants were moving (i.e., walking would be standing). Lying was used as the reference category, and the other postures were included as additional coefficients (see Model 2). The *pHR*, *VM*, $\beta_{0}$, $\beta_{1}$, i, and *ε* terms have the same meaning as introduced in Model 1. The new terms $P_{2}$ and $P_{3}$ are dummy variables that correspond to the two other postures (sitting and standing, respectively), whereas the terms $\beta_{2}$ and $\beta_{3}$ are the additional coefficients that represent the change in HR associated with these specific postures.

Model 2 (pHR ~ VM + posture):

$$\;{\mathrm{pHR}}_{i}=\beta_{0}+\beta_{1}\;{\mathrm{VM}}_{i}+\sum_{j=2\;}^{j=3}\left(\beta_{j}\;P_{i,j}\right)+\varepsilon_{i}.$$

In the third model, all seven activity categories were included as dummy variables: three static activities representing the three postures, as well as four dynamic activities representing the activity types. The first category, lying, was again taken as the reference category to avoid multicollinearity between dummy variables (hence, numbers 2 to 7 equals sitting, standing, walking, jogging, cycling, and walking stairs). $\beta_{0}$ therefore equals the intercept term that represents the expected HR during lying. The *pHR*, *VM*, $\beta_{1}$, i, and *ε* terms were already introduced in Model 1. The terms $A_{2}$, $A_{3}$, $A_{4}$, $A_{5}$, $A_{6},$ and $A_{7}$ represent dummy variables for the six non‐lying activities (sitting, standing, walking, jogging, cycling, and walking stairs, respectively), while the $\beta_{2}$, $\beta_{3}$, $\beta_{4}$, $\beta_{5}$, $\beta_{6,}$ and $\beta_{7}$ correspond to the changes in intercept associated with these activities.

Model 3 (pHR ~ VM + posture + activity type):

$${\mathrm{pHR}}_{i}=\beta_{0}+\beta_{1}\;{\mathrm{VM}}_{i}+\sum_{j=2\;}^{j=7}\left(\beta_{j}\;A_{i,j}\right)+\varepsilon_{i}.$$

In the fourth model, a lag function was incorporated into model 3 to consider the prolonged physiological effects of moderate‐to‐vigorous physical activity. This lag function contained the average VM during six preceding 30 s windows. The equation for the lag function contains the subscript “*t*” below and above the summation sign. This subscript represents the number of preceding 30 s windows that were included as predictors in the model. We selected a conservative lag length of six 30 s windows, totaling 3 min, to account for the prolonged impact of physical activity. Although it is not abnormal for moderate‐to‐vigorous physical activity to have an effect that lasts longer than 3 min, inclusion of a longer lag requires removing more observations at the beginning of the experiment (because you cannot compute the average VM in the preceding observations if there are not enough preceding observations). Therefore, we opted for a more conservative approach by including only six preceding windows, or 3 min, to predict the current HR. This decision specifically applies to the controlled laboratory setting used here. In an ambulatory context, one might consider incorporating more windows to better capture the prolonged impact of physical activity.

Model 4 (pHR ~ VM + posture + activity type + lagged VM):

$$\;{\mathrm{pHR}}_{i}=\beta_{0}+\beta_{1}\;{\mathrm{VM}}_{i}+\;\sum_{j=2\;}^{j=7}\left(\beta_{j}\;A_{i,j}\right)\;+\beta_{8}\;\left(\;\frac{1}{6}\;\sum_{\begin{array}{c} t=i-1\\ \; \end{array}}^{t=i-6}\left({\mathrm{VM}}_{t}\right)\;\right)+\varepsilon_{i}.$$

### 2.7 HR reactivity

HR reactivity serves as a critical measure to explore the physiological effects of various conditions. In this study, the reactivity is assessed through two distinct methods. The first method, commonly employed in laboratory settings, involves subtracting the average HR during a baseline condition from the oHR during an experimental condition. This method will henceforth be referred to as “classic heart rate reactivity.” The second method, aHR, involves subtracting the estimated movement‐related HR fluctuations from the oHR. This effectively isolates HR fluctuations that are considered to be due to mental stress. Condition means were obtained by averaging across all 30 s windows of the condition.

#### 2.7.1 Classic HR reactivity

Classic HR reactivity (ΔHR) was calculated for each 30 s window by subtracting the average HR during posture‐specific baselines from the HR observed during the conditions. Baselines were aligned with posture by selecting the baseline with corresponding posture at the beginning of the experiment (e.g., sitting baseline for mental stress tasks or standing baseline for walking). In the first study protocol, there were two baseline conditions per posture. The baseline HR per posture was calculated as the average of these two conditions. In the second study protocol, only one baseline condition per posture was available. The baseline HR per posture was calculated as the average of this one condition.

For periods in between conditions, when posture or activity type was unknown, matching posture‐specific baselines was not feasible. Consequently, to calculate ΔHR across all 30 s windows in these periods, the average HR within the same period was subtracted from the HR of each 30 s window. This procedure creates a continuous series of 30 s values across the entire experiment, which can be used for visualization. It is important to note that the ΔHR values during periods of unknown posture or activity type were only used for visualization purposes and were not included in the analyses.

#### 2.7.2 aHR

The aHR was calculated by subtracting the pHR from the oHR. In the framework of aHR, pHR represents the movement‐related HR fluctuations. The residual variation in HR that was not explained by physical activity, aHR, was considered indicative of mental stress.

aHR=oHR−pHR.

### 2.8 Model selection

We first selected the best model from the above models 1 to 4. This was done by testing whether the inclusion of each set of predictors (posture, activity type, and lagged version of VM) contributed to improved HR estimation performance. The average adjusted R‐squared (adjusted *R* ^2^) and the average root mean squared error (RMSE) were calculated for all four linear regression models of each participant, which served as metrics for explained variance and the accuracy of HR estimation. Stress conditions were excluded from this analysis because increases in HR due to mental stress conditions are not captured by the predictors that are solely related to physical activity and postural changes. Therefore, a mismatch between pHR and oHR is expected. We hypothesized that each subsequent model would show a significantly higher adjusted *R* ^2^ as well as a significantly lower RMSE. This hypothesis was tested using a paired *t* test, where the significance threshold was adjusted for testing multiple metrics (0.05/2 = 0.025). A *p*‐value < 0.025 would indicate the rejection of the null hypothesis, suggesting that the inclusion of this set of predictors yielded a significant improvement in HR estimation.

### 2.9 Model validation

The last model that yielded a significant improvement in adjusted *R* ^2^ as well as in RMSE of HR estimation was implemented for further analysis. The goal was to assess the effectiveness of aHR to control for physical activity and posture induced HR fluctuations, allowing for a better detection of instances of mental stress. The first step was to visualize the time series of oHR, pHR, classic HR reactivity (ΔHR) and aHR averaged over all participants in conjunction with experimental conditions. The ΔHR and aHR served similar conceptual functions: metrics to quantify the physiological response to distinct conditions. The key distinction is that ΔHR, unlike aHR, is not corrected for movement. Therefore, comparison of these two metrics could be employed to test our hypothesis that aHR is less sensitive to the impact of physical activity, but more sensitive to the impact of mental stress.

For conditions involving posture changes or physical activity, we anticipated strong agreement between pHR and oHR. As each subsequent model was enriched with additional predictors related to physical activity, pHR should closely follow oHR during posture changes and physical activity. On the contrary, we anticipated a discrepancy between ΔHR and aHR during physical activity due to the fact that ΔHR, unlike aHR, is not corrected for movement.

In mental stress conditions, we anticipated the opposite effect. If a mental stress condition were to elicit a physiological response (i.e., increased HR), we would expect a discrepancy between oHR and pHR. The physiological response is namely not of physical origin and its psychological origin cannot be captured by accelerometer signals. The discrepancy between oHR and pHR (aHR) should as a consequence be selectively increased during psychologically stressful conditions. This means in practical terms that aHR was expected to closely follow ΔHR during mental stress conditions.

#### 2.9.1 Between‐subject analysis

To explore the agreement between aHR and ΔHR in terms of detecting instances of mental stress, we generated scatterplots with the average aHR per participant plotted on the *x* axis and the average ΔHR per participant plotted on the *y* axis for each stress condition. ΔHR served as a benchmark for physiological reactivity to distinct conditions, as it is commonly employed in laboratory settings. The scatterplots incorporated an identity line, a center of mass, and the between‐subject correlation coefficient to visualize the agreement between the two methods. We anticipated a robust correlation, evident by a scatter cloud closely aligned to the identity line, with the center of mass closely situated near the identity line. This would imply a strong agreement between aHR and ΔHR in terms of quantifying individual differences in the physiological response to stressful conditions.

#### 2.9.2 Within‐subject analysis

We only included the larger second study (*n* = 128) for the within‐subject analysis, as the first study only had one short experimental stress condition and had no data regarding subjective experiences available. To investigate how well aHR can differentiate physical stress from mental stress within participants, we selected the highest 30% (equaling the average time spent in experimental stress conditions relative to total time spent in all experimental conditions) of aHR observations per participant as potential moments of mental stress. As we accounted for the impact of physical activity and postural changes on HR, these above‐threshold aHR moments should not reflect movement‐related HR fluctuations. Instead, these moments were expected to reflect HR responses to mental stress. The actual involvement of a participant in experimental stress conditions was considered the ground truth for mental stress exposure, whereas the involvement in any other experimental condition (baselines, postural changes, and physical activity) was considered the ground truth for non‐stress exposure. To assess whether experimental stress conditions generated subjective stress, we used self‐reported momentary affective states during baseline and directly after experimental stress conditions. These included three items of negative affect (insecure, anxious, and irritated) and three items of positive affect (relaxed, cheerful, and content).

For each participant, we established the overlap between moments identified by above‐threshold aHR and the ground truth using a classification approach within participants. A confusion matrix was computed per participant, with values proportional to the actual occurrence (number of positives in the ground truth) of the category. Subsequently, the confusion matrix for the aHR was averaged across all participants and presented in combination with the standard deviation across participants. A similar procedure was followed for the oHR, which is not corrected for physical activity or postural change. Again using a threshold of 30% highest HR to detect potential moments of stress, we again computed a confusion matrix. This allows for a comparison between the performance of aHR and the performance of observed (uncorrected) HR for the detection of mental stress. Several metrics were reported for both measures (aHR and oHR): F1, precision, and recall. We anticipated that high aHR moments should align with the ground truth for mental stress, more so than uncorrected HR. This would imply that aHR can more effectively differentiate between physical and mental stress.

## 3 RESULTS

### 3.1 Participants

Of the 200 participants who took part in one of the two study protocols, 3 had to be excluded from the data analysis entirely. Two participants were excluded due to incorrect settings for the accelerometer sampling frequency, while one participant had to be excluded due to a corrupt file. The remaining 197 participants (60% women and 40% men) had an average age of 22.6 years (SD = 4.7) and an average BMI of 23.6 kg/m^2^ (SD = 3.3). Two participants did not perform any of the mental stress conditions. Consequently, they were not included in the subsequent analysis that focused on reactivity during stressful conditions.

### 3.2 Data quality

The recording of the full experimental protocol lasted on average 120.5 min (SD = 15.7). Of this time, participants spent on average 61.5 min (SD = 5.2) in experimental conditions. The data quality was high, on average 0.8% (SD = 3.4) of 30 s windows had to be completely excluded due to lack of available HR data. Most of the time data were available with no or only a few heartbeats marked as artifacts that needed to be interpolated. On average, 0.47% (SD = 1.13%) of beats were interpolated per participant. There was no missingness at all for accelerometer data.

### 3.3 Activity recognition

**FIGURE 2:**
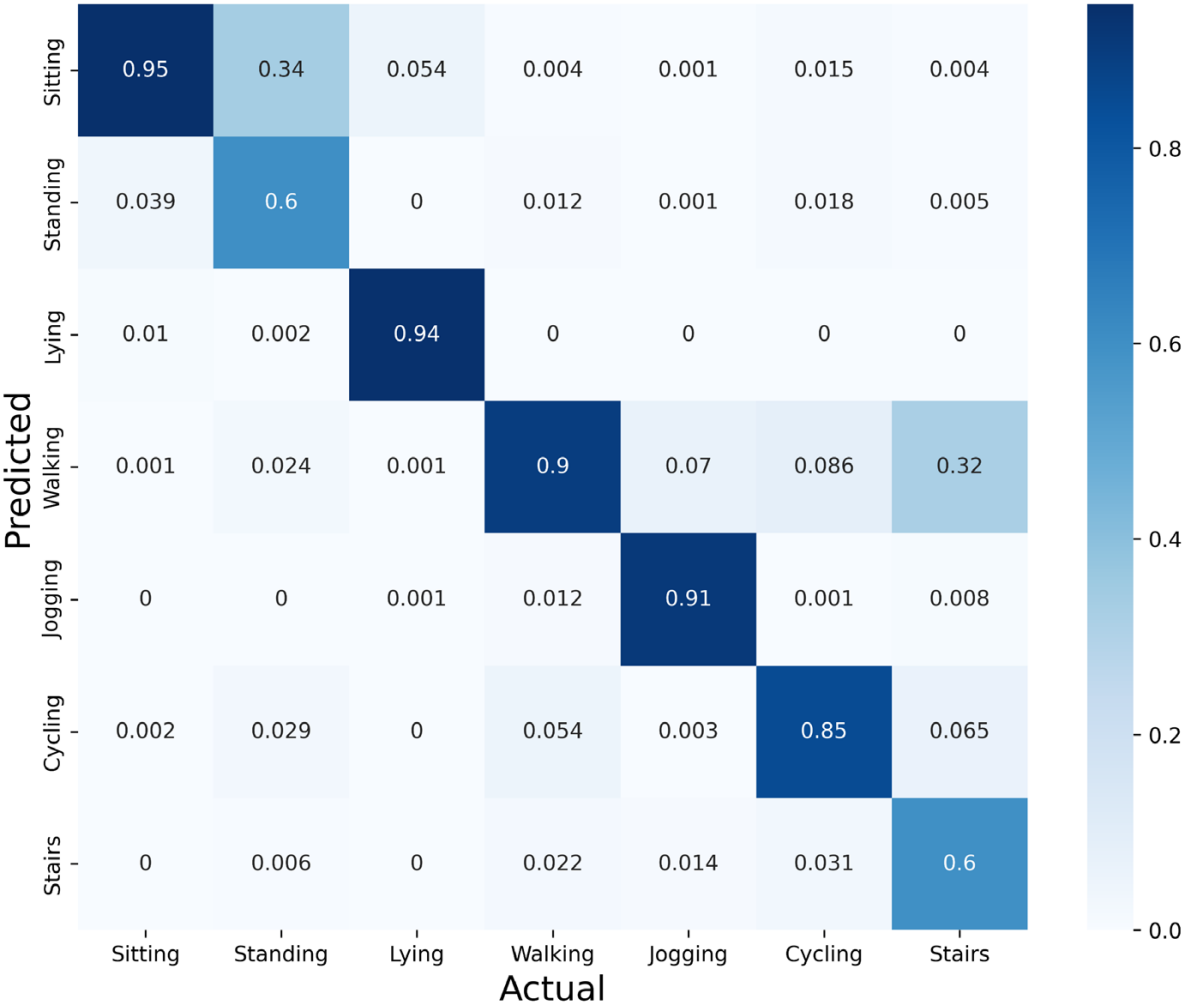
Confusion matrix containing the results of the activity recognition algorithm. All values are proportional to the actual occurrence (number of positives in the ground truth) of the category.

Figure 2 provides an overview of the activity classification results in the form of a confusion matrix. The predictions from the three classification algorithms (i.e. the SVM static‐dynamic classifier, XGBoost posture classifier, and CNN dynamic activity type classifier) contributed in stages (see Figure 1) to the final results presented here. The complete algorithm achieved an accuracy of 87.19% (SD = 6.83), a weighted F1 of 86.43% (SD = 7.19), a weighted precision of 87.87% (SD = 6.66), and a weighted recall of 87.19% (SD = 6.83).

The different steps of the algorithm showed some variability in terms of accuracy. The SVM static‐dynamic classifier achieved an accuracy of 98.48% (SD = 2.21), the XGBoost posture classifier achieved an accuracy of 88.49% (SD = 8.39), and the CNN dynamic activity type classifier had an accuracy of 85.04 (SD = 7.99).

### 3.4 Model selection

**FIGURE 3:**
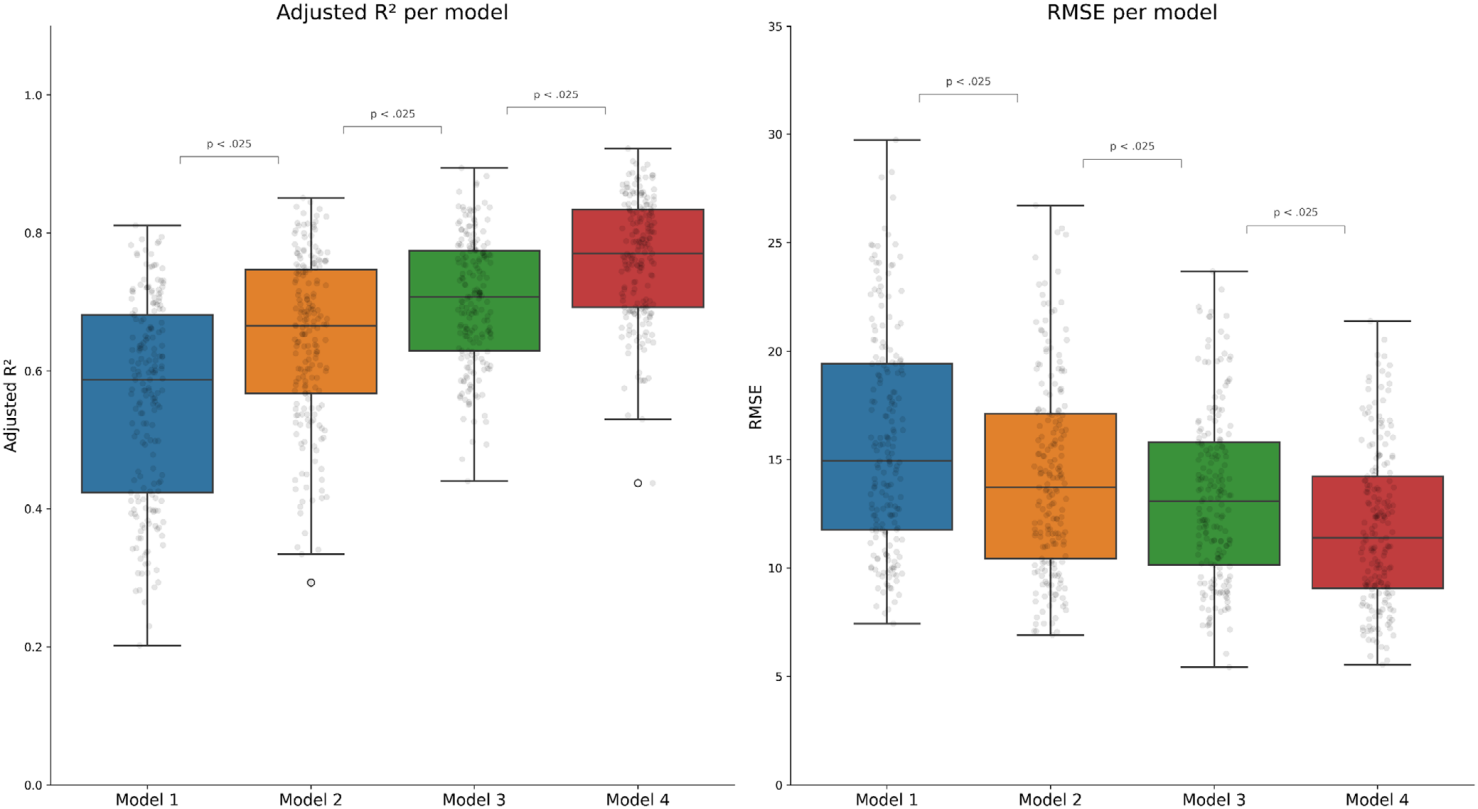
Adjusted *R* ^2^ and RMSE for each of the four models.

To evaluate the impact of including different sets of predictors (VM, posture, activity type, and lag function for recent VM) on HR estimation, we examined adjusted *R* ^2^ and RMSE for all four linear regression models of each participant. The results are shown in Figure 3. Each subsequent model exhibited a significantly higher adjusted *R* ^2^ (*p* < .025) and a significantly lower RMSE (*p* < .025).

Model 2 (*M* = 0.64, SD = 0.12) showed a significantly higher adjusted *R* ^2^ than model 1 (*M* = 0.56, SD = 0.15), *t*(196) = 17.8, *p* < .001. Model 3 (*M* = 0.70, SD = 0.09) had a significantly higher adjusted *R* ^2^ than model 2 (*M* = 0.64, SD = 0.12), *t*(196) = 14.3, *p* < .001. Model 4 (*M* = 0.76, SD = 0.09), compared to model 3 (*M* = 0.70, SD = 0.09), had significantly higher adjusted *R* ^2^, *t*(196) = 27.7, *p* < .001.

In terms of RMSE, model 2 (*M* = 14.21, SD = 4.49) demonstrated a significantly lower RMSE than model 1 (*M* = 15.78, SD = 5.01), *t*(196) = −17.1, *p* < .001. Similarly, model 3 (*M* = 13.26, SD = 3.93) had a significantly lower RMSE than model 2 (*M* = 14.21, SD = 4.49), *t*(196) = −11.6, *p* < .001. Finally, model 4 (*M* = 11.84, SD = 3.60), compared to model 3 (*M* = 13.26, SD = 3.93), had a significantly lower RMSE, *t*(196) = −24.5, *p* < .001.

In short, model 4 significantly outperformed the other three models. This result indicates an improvement in HR prediction following the incorporation of dummy variables for posture, dummy variables for activity type, and a lag function for recent movement intensity. These findings are consistent with our hypothesis that the inclusion of each set of predictors yielded a substantial improvement in HR estimation. The beta‐coefficients averaged across all participants for all parameters of all four models can be found in Table 2.

**TABLE 2:** The average beta‐coefficient (±standard deviation) across participants, per predictor per model.

	Model 1	Model 2	Model 3	Model 4
Intercept	86.5 (12.9)	69.8 (10.3)	70.8 (10.7)	69.1 (10.4)
Mean VM (g)	96.6 (25.3)	83.4 (22.3)	70.6 (38.2)	36.0 (31.9)
Lying (reference)				
Sitting		16.1 (10.6)	13.4 (10.4)	12.9 (8.6)
Standing		23.3 (10.8)	20.8 (10.9)	19.8 (9.9)
Walking			21.8 (13.2)	18.6 (12.2)
Jogging			30.1 (27.6)	22.6 (23.6)
Cycling			35.9 (16.8)	33.7 (16.8)
Stairs			37.1 (17.9)	34.3 (17.3)
Lag VM (g)				58.6 (23.9)

*Note*: From the second model onward, lying was used as the reference category to avoid multicollinearity between dummy variables.

### 3.5 Model validation

**FIGURE 4:**
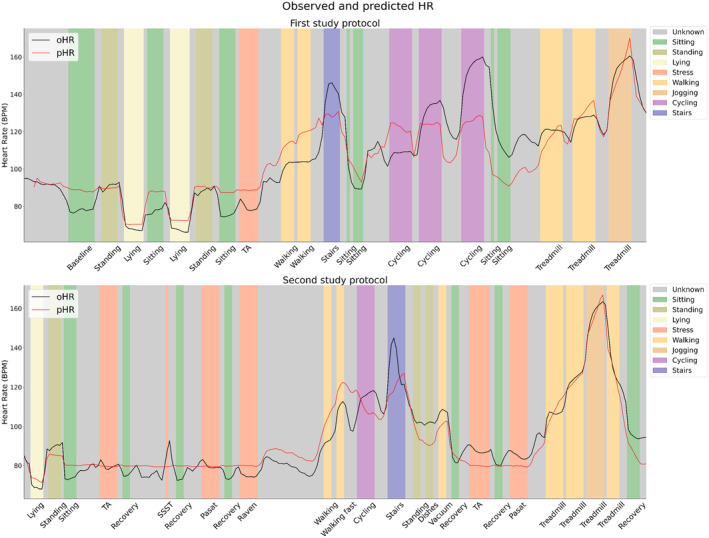
Overview of the observed and predicted heart rate averaged over all participants for both study protocols. The top plot shows the first study protocol, while the bottom plot shows the second study protocol. Vertical colored bars indicate the different categories present in the study protocol. The color coding is clarified in the external legend to the right of the plot.

**FIGURE 5:**
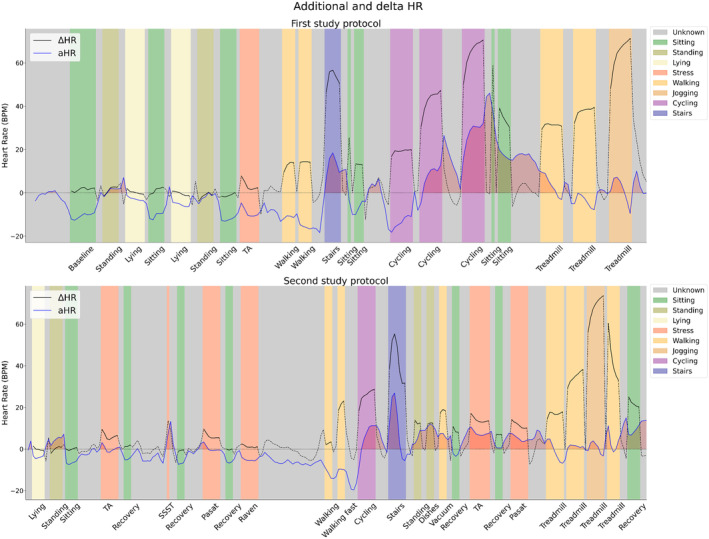
Overview of the additional heart rate, as well as the observed heart rate reactivity, averaged over all participants for both study protocols. The top plot shows the first study protocol, while the bottom plot shows the second study protocol. Vertical colored bars indicate the different categories present in the study protocol. The color coding is clarified in the external legend to the right of the plot.

Although the increase in adjusted *R* ^2^ and the decrease in RMSE are evident, these results do not elucidate the effectiveness of aHR in correcting for physical activity and postural change or its sensitivity in detecting instances of mental stress. To delve deeper into these aspects, a visual representation of the HR dynamics under varying experimental conditions was generated. Figure 4 presents the time series of oHR and pHR in combination with the experimental conditions as color‐coded vertical bars. Figure 5 presents the time series of aHR and ΔHR in combination with the experimental conditions as color‐coded vertical bars. Table 3 provides a detailed overview of the averages and standard deviations for aHR and ΔHR per experimental condition.

**TABLE 3:** The average ± standard deviation for each experimental condition in the two study protocols.

Condition	*n*	ΔHR (±SD)	aHR (±SD)	*r*
Study 1				
Baseline	70	1.74 (3.34)	−12.16 (6.13)	.39
Standing	69	1.22 (1.90)	−0.59 (6.53)	.23
Lying down	70	0.27 (1.20)	−2.69 (2.63)	.43
Sitting	70	1.19 (2.09)	−12.07 (6.42)	.32
Lying down	70	−0.30 (1.23)	−4.03 (5.48)	.22
Standing	70	−1.23 (1.95)	−3.06 (6.77)	.33
Sitting	70	−1.19 (2.06)	−14.04 (6.34)	.36
Tone Avoidance	68	2.60 (5.54)	−10.71 (6.05)	.54
Walking outside at leisurely pace	70	12.83 (10.52)	−12.32 (6.30)	.20
Walking outside while talking	69	14.25 (10.15)	−17.37 (7.55)	−.01
Walking stairs	69	52.40 (12.21)	11.85 (9.68)	.20
Recovery (sitting)	68	17.78 (11.38)	−4.47 (8.12)	.74
Ergometer 50 Watt with 60 RPM	68	23.38 (11.44)	−13.74 (9.27)	.16
Ergometer 100 Watt with 60 RPM	68	49.33 (16.46)	9.93 (8.32)	.66
Ergometer 150 Watt with 60 RPM	65	71.03 (15.09)	30.28 (6.75)	.54
Recovery (sitting)	68	41.33 (13.68)	23.38 (8.66)	.96
Treadmill (4.5–5 km/h)	68	32.25 (13.18)	5.04 (6.55)	.45
Treadmill (6–6.5 km/h)	67	39.82 (14.51)	−0.07 (6.64)	.16
Treadmill (7.5–8 km/h)	67	69.18 (15.76)	1.66 (5.91)	.12
(Recovery (sitting))	40	41.60 (13.48)	10.76 (5.02)	.63
(Vacuum cleaning)	23	30.31 (13.79)	6.21 (10.60)	.46
(Moving plates)	22	28.18 (14.80)	−1.35 (10.52)	.40
Study 2				
Lying down	125	0.00 (0.00)	−3.73 (4.31)	.00
Standing	125	0.00 (0.00)	3.98 (8.12)	−.06
Sitting	126	0.00 (0.00)	−6.68 (5.69)	.01
Tone Avoidance	126	6.73 (9.85)	0.61 (6.75)	.85
Recovery (sitting)	125	1.49 (5.31)	−4.82 (4.57)	.38
Short Sing‐a‐Song Stress Test	124	13.37 (14.00)	7.58 (10.95)	.93
Recovery (sitting)	126	−0.40 (6.23)	−6.64 (5.13)	.54
Paced Auditory Serial Addition Test	126	6.75 (10.53)	0.61 (7.65)	.87
Recovery (sitting)	126	−0.08 (5.89)	−6.18 (4.63)	.49
Raven's progressive matrices	125	1.55 (6.81)	−4.94 (4.36)	.62
Walking at natural pace	125	3.52 (11.98)	−11.40 (5.73)	−.03
Fast walking	125	21.68 (15.90)	−9.52 (7.01)	.13
Cycling	123	25.73 (20.43)	5.24 (7.44)	.67
Walking stairs	125	42.25 (18.55)	10.52 (7.65)	.31
Recovery (standing)	125	12.38 (13.65)	5.14 (8.21)	.58
Dish washing	125	11.92 (12.75)	11.18 (7.63)	.77
Vacuum cleaning	125	17.94 (14.83)	6.66 (7.33)	.36
Recovery (sitting)	126	8.85 (10.93)	−2.92 (7.66)	.82
Tone Avoidance	125	14.08 (13.29)	7.63 (9.90)	.93
Recovery (sitting)	125	6.86 (9.28)	0.41 (6.26)	.80
Paced Auditory Serial Addition Test	125	11.52 (11.44)	5.09 (8.24)	.89
Treadmill (4.5–5 km/h)	125	17.32 (14.09)	−0.91 (4.33)	.45
Treadmill (6–6.5 km/h)	125	34.71 (16.12)	1.16 (4.04)	.27
Treadmill (7.5–8 km/h)	120	66.80 (16.26)	0.66 (2.25)	.13
Treadmill (3.7–4 km/h)	121	41.93 (17.30)	2.79 (5.36)	−.00
Recovery (sitting)	123	21.27 (12.29)	8.22 (7.95)	.89

*Note*: *n* represents the number of participants with available data during the specific condition, and *r* represents the Pearson correlation coefficient between the averages per participant of ΔHR and aHR.

The time series of pHR and oHR showed strong overall resemblance. HR prediction was responsive to postural manipulation, which can be observed from the fact that pHR followed the trend of oHR after posture changes. For example, there is a decrease in pHR after lying down or an increase in pHR after standing up. However, the time series showed a consistent and substantial overestimation of pHR (and corresponding underestimation of aHR) while sitting. The aHR was on average −12.1 (SD = 6.4) beats per minute (BPM) and −14.0 (SD = 6.3) BPM during the two sitting conditions at the beginning of the first study protocol. We believe that this overestimation of HR during sitting can potentially be attributed to post‐exercise recovery conditions. Recovery conditions were characterized by an increased HR in the absence of movement captured by the accelerometer. This could lead to a higher baseline HR while sitting silently and a corresponding overestimation of HR during sitting conditions that were not directly following physical activity.

The validity of HR prediction during physical activity was also evident, as illustrated by the close alignment of pHR with oHR during most physical activities. However, the cycling conditions in the first study protocol exhibited a peculiarity with low aHR (on average − 13.7 ± 9.3 BPM) during the first block and high aHR (on average 30.3 ± 6.8 BPM) during the third block. The inability to consistently suppress the impact of cycling on HR is likely attributed to the fact that this condition was performed on an ergometer. The resistance of the ergometer was increased from 50 to 100 to 150 watts in three blocks, without adjustment of the number of revolutions per minute. Consequently, there was no increase in movement intensity associated with an increase in physical workload. The lag function to account for prolonged impact of physical activity was also found ineffective following the ergometer conditions because the increased physical workload was not properly captured by the accelerometer. This exemplifies the problem of distinct relationships between movement intensity and physical workload for various activity types. Other examples of conditions that proved difficult to suppress in terms of HR were walking stairs (average aHR of 11.9 ± 9.7 BPM and 10.5 ± 7.7 BPM for study 1 and study 2, respectively) and vacuum cleaning (average aHR of 6.2 ± 10.6 BPM and 6.7 ± 7.3 BPM for study 1 and study 2, respectively).

#### 3.5.1 Between‐subject analysis

**FIGURE 6:**
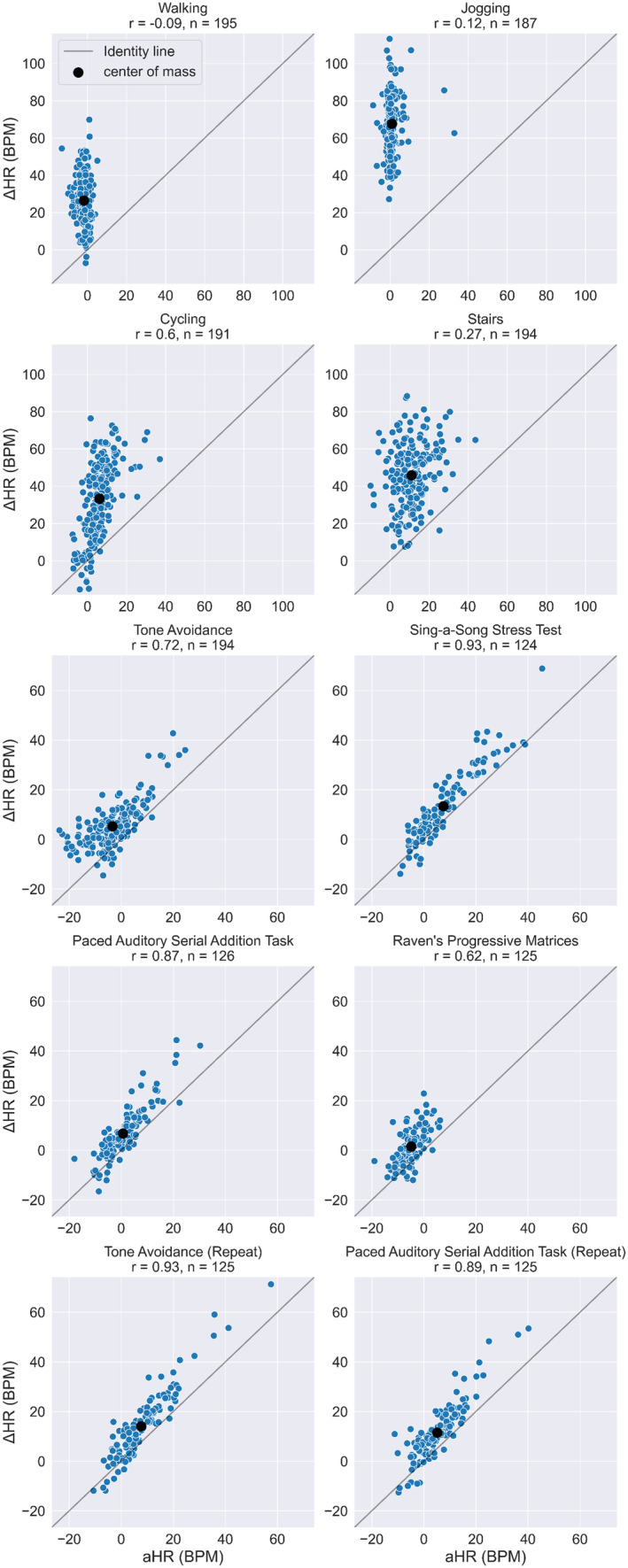
Scatter plot with aHR on the *x* axis and ΔHR on the *y* axis. The blue dots represent average ΔHR and aHR per participant in the specific conditions. Top four plots represent physical activity conditions, and have a wider scale than the mental stress conditions in the bottom six plots. The number of participants per condition is indicated in the title above all separate plots, as well as the correlation coefficient. The diagonal line represents the identity line, and the larger black dot represents the center of mass.

To explore the agreement between aHR and ΔHR in terms of detecting instances of mental stress, scatterplots were created and presented in Figure 6. Scatterplots showed a discrepancy between aHR and ΔHR in terms of baseline levels, as can be seen from the center of mass located above the identity line. This is consistent with the abovementioned underestimation of HR during sitting conditions. The correlation between aHR and ΔHR for all stress conditions separately ranged from 0.62 to 0.93. The lowest correlation of 0.62 was found for the Raven's progressive matrices condition. Interestingly, this condition elicited the smallest physiological response with an average ΔHR of 1.6 (SD = 6.8) BPM. The correlation was highest (0.93) during the short sing‐a‐song stress test (average ΔHR of 13.4 ± 14.0 BPM) and during the second tone avoidance condition (average ΔHR of 14.1 ± 13.3 BPM), the conditions that most frequently evoked strong physiological stress responses. The high correlation coefficients during the mental stress conditions indicated the ability of aHR to detect between‐subject variability in physiological responses to mental stress. However, during many conditions, in particular, in study protocol 1, aHR produced negative values.

#### 3.5.2 Within‐subject analysis

**FIGURE 7:**
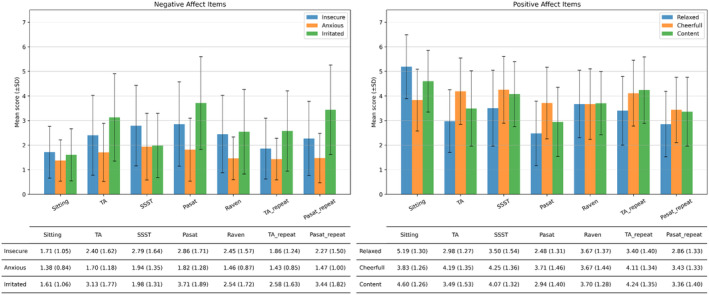
Manipulation check displaying the average and standard deviation per item of negative affect and positive affect.

The manipulation check to assess if mental stress conditions generated changes in self‐reported affective states is shown in Figure 7. The results indicated that all three negative affect items were on average consistently higher following an experimental stress condition relative to baseline. Two positive affect items, relaxed and content, were on average consistently lower following an experimental stress condition relative to baseline. The last item of positive affect, cheerful, did not show a consistent increase or decrease in response to mental stress conditions relative to baseline.

**FIGURE 8:**
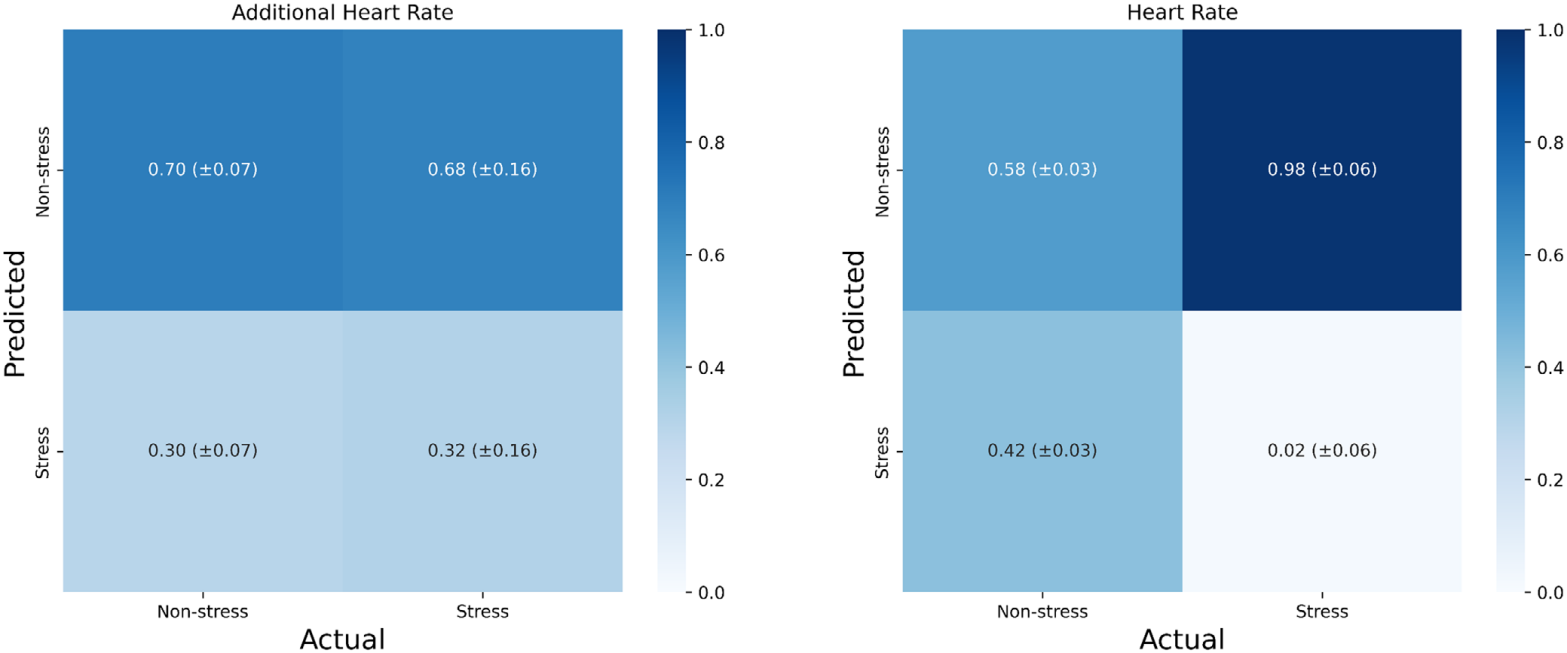
Confusion matrix of the within‐subject model validation analysis. All values are calculated within participants and subsequently transformed to be proportional to the actual occurrence (number of positives in the ground truth) of the category. Then, the values in the confusion matrix are averaged across participants and presented in combination with the standard deviation across participants between brackets. The left panel indicates the classification approach executed with additional heart rate, whereas the right panel indicates the classification approach executed with the observed heart rate.

To investigate how well aHR can differentiate physical stress from mental stress within participants, we examined the overlap between moments identified by high aHR and the ground truth (operationalized as actual involvement in experimental stress conditions) using a classification approach. The confusion matrix averaged across all participants is reported in Figure 8. The confusion matrix indicated that on average 32% of all observations in mental stress conditions were classified as mental stress based on above‐threshold aHR (equaling a recall score of 0.32, SD = 0.16). Out of the observations predicted as mental stress from aHR, only 31% were actually coming from one of the four experimental mental stress conditions (as indicated by a precision score of 0.31, SD = 0.16). The stress category had an average F1 of 0.31 (SD = 0.16). In contrast, using the observed (uncorrected) HR, only 2% of all observations in experimental stress conditions were classified as mental stress (recall of 0.02, SD = 0.06). Mental stress predicted from above‐threshold oHR had a very low F1 of 0.02 (SD = 0.06) and precision of 0.02 (SD = 0.07). Moreover, aHR performed reasonably well at avoiding false alarms due to physical activity, with 70% (SD = 7%) of all observations in other experimental conditions (baselines, postural changes, or physical activity) correctly classified as non‐stress based on aHR. In comparison, the oHR labeled 58% (SD = 3%) of all observations in other experimental conditions (baselines, postural changes, or physical activity) correctly as non‐stress.

## 4 DISCUSSION

The primary objective of this study was to develop a novel method to estimate the suprametabolic HR or “additional heart rate” using accelerometers. We argued that the estimation of movement‐related HR fluctuations can be improved by taking posture, activity type, and concurrent as well as recent movement intensity into account (Altini et al., 2015; Bonomi et al., 2009; Kusserow et al., 2013; Yang et al., 2017). In pursuit of this objective, we developed a machine learning algorithm to classify posture and activity type from triaxial accelerometer signals across a range of postures and activity types in a controlled laboratory environment. The classification results indicated an overall accuracy across all seven categories of 87.2%. Subsequently, it was tested whether the addition of dummy variables for posture, dummy variables for activity type, and a lag function for recent movement intensity led to a significant improvement in HR estimation during this laboratory experiment. The results showed that adding these predictors led to a significant increase in adjusted *R* ^2^ and a significant decrease in RMSE. The average adjusted *R* ^2^ increased from 0.56 to 0.76 (+36%), whereas the average RMSE decreased from 15.78 to 11.84 (−25%). Further analysis indicated that the model including predictors for posture, activity type, and recent movement intensity was indeed sensitive to postural manipulation and physical activity. However, there was a consistent and substantial overestimation of HR while sitting at rest, and HR in certain activity types proved challenging to predict correctly. Despite these challenges, aHR showed strong agreement with classic HR reactivity on a between‐subject level, with correlations ranging from 0.62 to 0.93 across six mental stress conditions. Furthermore, aHR outperformed observed heart rate in detecting exposure to mental stress conditions on a within‐subject level. Even so, separation of physical stress from mental stress within participants was modestly successful, for instance, shown by the fact that on average only 32% of observations in experimental stress conditions were classified as mental stress based on aHR, and that on average 30% of observations in other experimental conditions (baselines, postural changes, or physical activity) were misclassified as mental stress.

The overall activity classification accuracy of 87.2% aligns well with comparable studies conducted in this domain. Altini et al. (2015) achieved an overall accuracy of 85% with one accelerometer placed on the thigh, whereas Coelho et al. (2022) achieved an overall accuracy of 88.4% with one accelerometer placed at the waist. It is worth highlighting that two categories proved challenging for accurate classification: standing was frequently misclassified as sitting, and walking stairs was frequently misclassified as walking. The confusion between standing and sitting parallels observations by Coelho et al. (2022), indicating a common difficulty in distinguishing sitting from standing with a waist‐ and hip‐worn accelerometer.

In the broader context of activity recognition algorithms, our performance falls below current benchmark algorithms (Ignatov, 2018; Su et al., 2019; Tufek et al., 2020; Wang et al., 2019) that achieve accuracies as high as 97%. However, these algorithms either integrated additional signals such as gyroscopes (Tufek et al., 2020; Wang et al., 2019) or had a narrower range of categories to be classified (Ignatov, 2018; Su et al., 2019). Furthermore, as emphasized by Coelho et al. (2022), these algorithms typically rely on complex networks that require substantial computational resources, making them less suitable for embedded solutions. The ability to perform real‐time classification of postures and activity types would also facilitate real‐time computation of periods of aHR. This could provide opportunities for the implementation of aHR in triggering just‐in‐time adaptive interventions in ambulatory settings (Nahum‐Shani et al., 2017; Schwerdtfeger & Rominger, 2021).

A direct comparison of the improvement in adjusted *R* ^2^ and RMSE with other studies that estimated the physiological impact of physical activity using accelerometers is complicated for three reasons. First, no previous studies have included all three predictors (i.e., posture, activity type, and recent movement intensity). Second, no comparison of RMSE between studies is feasible because these studies did not employ HR as outcome measure. Instead, studies reported alternative measures such as kcal/min (Altini et al., 2015), MJ/day (Bonomi et al., 2009), or HR variability (Brown et al., 2018; Verkuil et al., 2016). Third, no comparison of adjusted *R* ^2^ between studies is feasible because data collection of most other studies took place in an ambulatory setting (Altini et al., 2015; Bonomi et al., 2009; Brown et al., 2018; Myrtek, 2004). The extent to which a laboratory protocol accurately reflects ambulatory measurements in terms of physical activity levels is uncertain. For example, Bonomi et al. (2009) estimated that participants in their ambulatory study spent an average of 75.6% of their time in sedentary activities, of which lying claimed 36.1%. In contrast, participants in our laboratory protocol were predicted to spend on average 65.9% (SD = 8.6) of their time sedentarily, and 4.8% (SD = 3.8) of their time involved lying. More strikingly, 42.3% (SD = 6.8) of the time in experimental conditions involved moderate‐ to vigorous‐intensity physical activity (MVPA). In comparison, a meta‐analysis that included 44,370 participants reported that the time spent in MVPA in daily life varied between nine cohorts from 0.8% to 3.9% (Ekelund et al., 2020). Substantial differences between studies in physical activity levels or range of activity types complicate direct comparison of the estimation of HR using accelerometers.

The increase in adjusted *R* ^2^ and the decrease in RMSE for our last model clearly confirm that current HR is sensitive to more than just ongoing physical activity; postural manipulation, activity type, and past physical activity also contribute to the current HR. However, we noticed some caveats in the estimation of movement‐related HR. First, even when using the best model with posture and lagged movement intensity, there was a consistent overestimation of HR while sitting at rest. This overestimation may partially be attributed to post‐exercise recovery conditions. Post‐exercise recovery conditions are marked by an elevated HR while there is no movement as recorded by the accelerometer, resulting in a heightened HR intercept across all conditions where participants were sitting at rest. Notably, an overestimated HR during sitting conditions leads to an underestimated aHR, as this is computed by subtracting the pHR from the oHR. Second, we encountered challenges in estimating HR during certain activity types, like cycling on an ergometer. This was probably attributed to the fact that this activity pairs a near absence of change in movement intensity with a strong increase in physical workload. As we use movement intensity as the proxy for physical workload, we fail to correctly model the typical linear relationship between physical workload and HR during bicycle ergometry. Enriching models with interaction terms between movement intensity and HR per activity type might be beneficial for estimating movement‐related HR for certain activity types. However, it would not have addressed this specific issue, as movement intensity remained non‐predictive for HR across various ergometer blocks. This underscores the persistent challenge posed by activity‐specific relationships between movement intensity and HR.

Aligned to the underestimation of aHR in baseline sitting conditions, we also observed an underestimation of aHR in comparison with ΔHR during mental stress conditions. This overall underestimation of aHR, for instance, evident by a 12 BPM underestimation during baseline sitting conditions in the first study protocol, is not an idiosyncrasy inherent in our newly proposed model. We also encounter this underestimation in the first model that “classically” only included movement intensity as a predictor. This model revealed an even more pronounced underestimation by aHR (data not shown), suggesting a more general problem rather than technical aspects of the proposed new method. A more likely cause of the underestimation is the experimental protocols used, in particular the lack of appropriate and prolonged baseline recordings.

### 4.1 Challenges in obtaining a baseline HR in controlled laboratory environments

This study examined the efficacy of aHR in a controlled laboratory environment. Controlled laboratory environments offer the advantage of precisely tracking posture and physical activity at each moment, which is essential for constructing a custom activity recognition algorithm. Furthermore, precise details on posture and activity type help provide information on how aHR behaves under specific postures or activity types. We repurposed existing data sets of laboratory protocols that, although not originally designed to investigate this research question, possessed distinct advantages. Both experimental protocols were extensive and incorporated a wide range of physically and psychologically demanding conditions. By combining both experimental protocols, we were able to report on a large sample size and reduced the likelihood of reporting results that are biased by the characteristics of the laboratory protocol such as the order of the experimental conditions. However, both protocols were characterized by a relative enrichment of physical activity conditions, evident by the large proportion of MVPA. Relatively short baselines were used and many were more reflective of recovery than true baselines. Moreover, baseline HR in laboratory setting can be shifted due to factors such as anticipatory tension or awareness of being observed. Therefore, the absence of a repeated and prolonged “vanilla” baseline (Jennings et al., 1992) may have biased the intercept of the HR prediction regression and led to a general underestimation of aHR.

### 4.2 Between‐subject and within‐subject detection of stress by aHR

On a between‐subject level, we found between‐subject correlations for aHR and ΔHR ranging from 0.62 to 0.93, despite the systematic underestimation of aHR. This suggests a good agreement in the ranking of participants as low‐ or high‐stress reactors using either aHR or “classic” HR reactivity. The correlation was particularly evident in stress conditions with high observed reactivity, highlighting the sensitivity of our approach to between‐subject variability in physiological responses to experimental stress conditions. Nonetheless, the underestimation of aHR poses a potential challenge to utilize an absolute cut‐off for detecting instances of mental stress in situations where ground truth is unknown, for instance, in ambulatory recordings (e.g., the highest 30% aHR or aHR above two times the standard error).

On a within‐subject level, we examined the overlap between moments identified by high aHR and the ground truth (operationalized as actual involvement in mental stress conditions) using a classification approach. The results indicated that aHR outperformed the oHR in detecting exposure to mental stress. Even so, partially due to the general underestimation of aHR, there was a high proportion of false negatives. On average, 68% (SD = 16%) of observations in experimental stress conditions were classified as not stressful based on aHR. This indicates that improving aHR estimation by adding longer baseline recording is needed, in order to overcome the underestimation of aHR. In addition, our conditions may not have induced sufficient levels of stress, or done so only in a subset of participants. The mental stress conditions exerted only a small effect on participants' affective states. Therefore, it might be overly simplistic to assume that our mental stress manipulations systematically evoked noticeable HR responses at all observations in all participants.

### 4.3 Application of aHR for detecting mental stress in ambulatory recordings

The remaining question is how this new method for aHR estimation would perform beyond the confined parameters of laboratory experiments. Ambulatory assessment in daily life comes with the disadvantages of unsupervised recordings, prominently including an absence of the perfect ground truth for posture and activity type that we have in the laboratory. Hence, creating a new classification model to predict posture and activity type from accelerometer signals outside the laboratory is not feasible. A reasonable assumption is that the models we created in laboratory settings should also hold in daily life. All predictions in the present study were namely performed on unseen data, due to the LOSO‐CV framework. This provides an indication of the generalizability of these classification models. However, whether this also extends to aHR estimation from these predicted postures and activity types remains an open question. An added challenge is that it is not easy to verify the aHR against a ground‐truth measure of stress reactivity, such as the task‐baseline reactivity employed here. In ambulatory setting, we have to rely on reported affective states or contextual information to know something about potential exposure to stressors.

Fortunately, these disadvantages are counterbalanced by clear advantages of ambulatory recordings. First, the ecological validity of naturally occurring stressors surpasses that of scripted stress conditions with fixed durations commonly encountered in laboratory settings. This could explain why exposure to stressors in naturalistic setting has been found to elicit a greater HR reaction than stressors in laboratory setting (Zanstra & Johnston, 2011). Larger HR reactivity in daily life may increase the sensitivity of our aHR method. Second, compared to laboratory experiments, the likelihood of capturing a more representative measurement of baseline HR is much larger in ambulatory recordings. To substantiate this statement, we extracted the average baseline HR for 174 participants of this laboratory protocol that also had data available for at least 8 h during an ambulatory recording. The baseline HR was defined as the lowest average HR during 10 consecutive minutes while being awake. A paired *t* test revealed that the average baseline HR was significantly lower in ambulatory recordings (*M* = 62.0, SD = 8.4) in comparison with laboratory recordings (*M* = 73.8, SD = 11.3), *t*(173) = −15.3, *p* < .001. This difference is very close to the offset we see for the aHR compared to the ΔHR and bodes well for the application of our new method in daily life.

### 4.4 Generalizability to different device placements

The present study was a proof of concept to examine whether the addition of predictors for posture, activity type, and (lagged) movement intensity can improve the estimation of the effects of movement on HR. This study utilized an accelerometer that was placed at the hip. Although our trained activity recognition algorithms will not hold for accelerometers with other placements, the concept of movement profiling for computing aHR should generalize to other placements. This would require training custom activity recognition algorithms that are specific to the device placement. The optimal placement of accelerometers for discerning posture or activity type remains a topic of debate. Findings are heterogeneous across various studies, with some suggesting chest and waist are the most suitable placement for discerning posture or activity type (Gjoreski et al., 2011), whereas other studies suggest that the thigh (Fortune et al., 2014) or the hip (Cleland et al., 2013) are the most suitable placements. It is important to recognize that no single placement is perfect, as each has its own caveats. On the one hand, as further corroborated by our findings, hip placement is less suitable to differentiate between sitting and standing (Coelho et al., 2022; Gjoreski et al., 2011). On the other hand, more distal placements (such as the wrist) can result in heterogeneous accelerometer recordings even within the same posture. For instance, a wrist‐worn accelerometer may capture different device orientations while sitting, depending on whether the arms are hanging vertically beside the body or resting horizontally on a surface, such as during desk work. In terms of estimating movement intensity, a proximal placement makes an accelerometer more sensitive to gross motor movements that involve all large muscle groups in comparison with a distal placement, as the sensor is closer to the center of mass (Yang & Hsu, 2010). This is a notable advantage of the device placement employed in this study, particularly when accounting for the physiological effects of physical activity.

## 5 CONCLUSION

In summary, this study found that the estimation of aHR using accelerometers can be improved by incorporating predictors for posture, activity type, and a lag function for recent physical activity. However, based on the application in our laboratory experiment, two caveats should be considered. That is, HR estimation in certain activity types remains challenging and HR while sitting at rest was consistently overestimated due to a protocol enriched for physical activity. The overestimated HR also manifested as a negative offset of aHR. Yet, although this offset poses a potential challenge when utilizing an absolute cut‐off for detecting instances of mental stress, we found good agreement between aHR and classic HR reactivity with between‐subject correlations ranging from 0.62 to 0.93 across stress conditions. These robust between‐subject correlations underscore the potential of our method. Lastly, while aHR improved differentiation between physical and mental stress in comparison with uncorrected HR on a within‐subject level, its ability to effectively classify mental stress in this laboratory protocol was modest. Future research should apply this new method in ambulatory assessment to explore how a more representative baseline HR and potentially higher physiological reactivity affect the usefulness of aHR in detecting instances of mental stress in ambulatory setting. We believe that this has the potential to advance the field of psychophysiological research at large, as the benefits of being able to differentiate between the impact of physical and mental demands on HR in daily life are evident.

## AUTHOR CONTRIBUTIONS

**Sjors R. B. van de Ven:** Data curation; formal analysis; methodology; visualization; writing – original draft. **Martin J. Gevonden:** Conceptualization; investigation; methodology; supervision; writing – review and editing. **Matthijs L. Noordzij:** Conceptualization; methodology; writing – review and editing. **Eco J. C. de Geus:** Conceptualization; funding acquisition; investigation; methodology; writing – review and editing.

## FUNDING INFORMATION

This article was written as part of the research project “Stress in Action”: www.stress‐in‐action.com. Stress in Action is financially supported by the Dutch Research Council and the Dutch Ministry of Education, Culture and Science (NWO gravitation grant number 024.005.010).

## CONFLICT OF INTEREST STATEMENT

The authors declare no competing interests.

## Data Availability

Data are available upon reasonable request.

## References

[psyp14721-bib-0001] Altini, M. , Penders, J. , Vullers, R. , & Amft, O. (2015). Estimating energy expenditure using body‐worn accelerometers: A comparison of methods, sensors number and positioning. IEEE Journal of Biomedical and Health Informatics, 19(1), 219–226. 10.1109/JBHI.2014.2313039 24691168

[psyp14721-bib-0002] Baldi, J. C. , Lalande, S. , Carrick‐Ranson, G. , & Johnson, B. D. (2007). Postural differences in hemodynamics and diastolic function in healthy older men. European Journal of Applied Physiology, 99(6), 651–657. 10.1007/s00421-006-0384-5 17226061

[psyp14721-bib-0003] Bergstra, J. , Yamins, D. , & Cox, D. (2013). *Hyperopt: A python library for optimizing the hyperparameters of machine learning algorithms* (pp. 13–19). 10.25080/Majora-8b375195-003

[psyp14721-bib-0004] Blix, A. S. , Stromme, S. B. , & Ursin, H. (1974). Additional heart rate—An indicator of psychological activation. Aerospace Medicine, 45(11), 1219–1222. 4429063

[psyp14721-bib-0005] Bonomi, A. G. , Plasqui, G. , Goris, A. H. C. , & Westerterp, K. R. (2009). Improving assessment of daily energy expenditure by identifying types of physical activity with a single accelerometer. Journal of Applied Physiology (Bethesda, MD: 1985), 107(3), 655–661. 10.1152/japplphysiol.00150.2009 19556460

[psyp14721-bib-0006] Brouwer, A.‐M. , van Dam, E. , van Erp, J. B. F. , Spangler, D. P. , & Brooks, J. R. (2018). Improving real‐life estimates of emotion based on heart rate: A perspective on taking metabolic heart rate into account. Frontiers in Human Neuroscience, 12, 284. 10.3389/fnhum.2018.00284 30061818 PMC6054929

[psyp14721-bib-0007] Brown, S. B. R. E. , Brosschot, J. F. , Versluis, A. , Thayer, J. F. , & Verkuil, B. (2018). New methods to optimally detect episodes of non‐metabolic heart rate variability reduction as an indicator of psychological stress in everyday life. International Journal of Psychophysiology, 131, 30–36. 10.1016/j.ijpsycho.2017.10.007 29055696

[psyp14721-bib-0008] Burg, M. M. , Schwartz, J. E. , Kronish, I. M. , Diaz, K. M. , Alcantara, C. , Duer‐Hefele, J. , & Davidson, K. W. (2017). Does stress result in you exercising less? Or does exercising result in you being less stressed? Or is it both? Testing the Bi‐directional stress‐exercise Association at the Group and Person (N of 1) level. Annals of Behavioral Medicine: A Publication of the Society of Behavioral Medicine, 51(6), 799–809. 10.1007/s12160-017-9902-4 28290065 PMC5597451

[psyp14721-bib-0009] Carroll, D. , Rick Turner, J. , & Hellawell, J. C. (1986). Heart rate and oxygen consumption during active psychological challenge: The effects of level of difficulty. Psychophysiology, 23(2), 174–181. 10.1111/j.1469-8986.1986.tb00613.x 3704073

[psyp14721-bib-0010] Chen, T. , & Guestrin, C. (2016). XGBoost: A scalable tree boosting system. In Proceedings of the 22nd ACM SIGKDD international conference on knowledge discovery and data mining (pp. 785–794). 10.1145/2939672.2939785

[psyp14721-bib-0011] Chollet, F. , & Others . (2015). Keras. *Github*. https://github.com/fchollet/keras

[psyp14721-bib-0222] Cleland, I. , Kikhia, B. , Nugent, C. , Boytsov, A. , Hallberg, J. , Synnes, K. , McClean, S. & Finlay, D. (2013). Optimal Placement of Accelerometers for the Detection of Everyday Activities. Sensors, 13(7), 9183–9200. 10.3390/s130709183 23867744 PMC3758644

[psyp14721-bib-0012] Coelho, Y. L. , Nguyen, B. , Santos, F. A. , Krishnan, S. , & Bastos‐Filho, T. F. (2022). A lightweight model for human activity recognition based on two‐level classifier and compact CNN model. In T. F. Bastos‐Filho , E. M. de Oliveira Caldeira , & A. Frizera‐Neto (Eds.), XXVII Brazilian congress on biomedical engineering (pp. 1895–1901). Springer International Publishing. 10.1007/978-3-030-70601-2_276

[psyp14721-bib-0013] Cohen, S. , Janicki‐Deverts, D. , & Miller, G. E. (2007). Psychological stress and disease. JAMA, 298(14), 1685–1687. 10.1001/jama.298.14.1685 17925521

[psyp14721-bib-0014] Cornelissen, V. A. , Verheyden, B. , Aubert, A. E. , & Fagard, R. H. (2010). Effects of aerobic training intensity on resting, exercise and post‐exercise blood pressure, heart rate and heart‐rate variability. Journal of Human Hypertension, 24(3), 175–182. 10.1038/jhh.2009.51 19554028

[psyp14721-bib-0015] de Geus, E. J. C. , & Gevonden, M. (2023). Acquisition and analysis of ambulatory autonomic nervous system data. In M. Mehl , E. Eid , C. Wrzus , J. Harari , & U. Ebner‐Priemer (Eds.), Mobile sensing in psychology: Methods and applications (pp. 129–167). Guilford Publications, Inc.

[psyp14721-bib-0016] de Geus, E. J. C. , Willemsen, G. H. M. , Klaver, C. H. A. M. , & van Doornen, L. J. P. (1995). Ambulatory measurement of respiratory sinus arrhythmia and respiration rate. Biological Psychology, 41(3), 205–227. 10.1016/0301-0511(95)05137-6 8608201

[psyp14721-bib-0017] Delistraty, D. A. , Greene, W. A. , Carlberg, K. A. , & Raver, K. K. (1991). Use of graded exercise to evaluate physiological hyperreactivity to mental stress. Medicine & Science in Sports & Exercise, 23(4), 476–481. 2056906

[psyp14721-bib-0018] Ebner‐Priemer, U. W. , Welch, S. S. , Grossman, P. , Reisch, T. , Linehan, M. M. , & Bohus, M. (2007). Psychophysiological ambulatory assessment of affective dysregulation in borderline personality disorder. Psychiatry Research, 150(3), 265–275. 10.1016/j.psychres.2006.04.014 17321599

[psyp14721-bib-0019] Edgell, H. , Robertson, A. D. , & Hughson, R. L. (2012). Hemodynamics and brain blood flow during posture change in younger women and postmenopausal women compared with age‐matched men. Journal of Applied Physiology (Bethesda, MD: 1985), 112(9), 1482–1493. 10.1152/japplphysiol.01204.2011 22362404

[psyp14721-bib-0020] Ekelund, U. , Tarp, J. , Fagerland, M. W. , Johannessen, J. S. , Hansen, B. H. , Jefferis, B. J. , Whincup, P. H. , Diaz, K. M. , Hooker, S. , Howard, V. J. , Chernofsky, A. , Larson, M. G. , Spartano, N. , Vasan, R. S. , Dohrn, I.‐M. , Hagströmer, M. , Edwardson, C. , Yates, T. , Shiroma, E. J. , … Lee, I.‐M. (2020). Joint associations of accelero‐meter measured physical activity and sedentary time with all‐cause mortality: A harmonised meta‐analysis in more than 44 000 middle‐aged and older individuals. British Journal of Sports Medicine, 54(24), 1499–1506. 10.1136/bjsports-2020-103270 33239356 PMC7719907

[psyp14721-bib-0021] Epel, E. S. , Crosswell, A. D. , Mayer, S. E. , Prather, A. A. , Slavich, G. M. , Puterman, E. , & Mendes, W. B. (2018). More than a feeling: A unified view of stress measurement for population science. Frontiers in Neuroendocrinology, 49, 146–169. 10.1016/j.yfrne.2018.03.001 29551356 PMC6345505

[psyp14721-bib-0022] Fortune, E. , Lugade, V. A. , & Kaufman, K. R. (2014). Posture and movement classification: The comparison of tri‐axial accelerometer numbers and anatomical placement. Journal of Biomechanical Engineering, 136(5). 10.1115/1.4026230 PMC4023813 24337255

[psyp14721-bib-0023] Gjoreski, H. , Lustrek, M. , & Gams, M. (2011). Accelerometer placement for posture recognition and fall detection. Seventh International Conference on Intelligent Environments, 2011, 47–54. 10.1109/IE.2011.11

[psyp14721-bib-0024] Gliner, J. A. , Bedi, J. F. , & Horvath, S. M. (1979). Somatic and non‐somatic influences on the heart: Hemodynamic changes. Psychophysiology, 16(4), 358–362. 10.1111/j.1469-8986.1979.tb01476.x 461664

[psyp14721-bib-0025] Grossman, P. , Wilhelm, F. H. , & Spoerle, M. (2004). Respiratory sinus arrhythmia, cardiac vagal control, and daily activity. American Journal of Physiology. Heart and Circulatory Physiology, 287(2), H728–H734. 10.1152/ajpheart.00825.2003 14751862

[psyp14721-bib-0026] Halliwill, J. R. , Buck, T. M. , Lacewell, A. N. , & Romero, S. A. (2013). Postexercise hypotension and sustained postexercise vasodilatation: What happens after we exercise? Experimental Physiology, 98(1), 7–18. 10.1113/expphysiol.2011.058065 22872658

[psyp14721-bib-0027] Halliwill, J. R. , Taylor, J. A. , Hartwig, T. D. , & Eckberg, D. L. (1996). Augmented baroreflex heart rate gain after moderate‐intensity, dynamic exercise. The American Journal of Physiology, 270(2 Pt 2), R420–R426. 10.1152/ajpregu.1996.270.2.R420 8779874

[psyp14721-bib-0028] Healey, J. , Nachman, L. , Subramanian, S. , Shahabdeen, J. , & Morris, M. (2010). Out of the lab and into the fray: Towards modeling emotion in everyday life. In P. Floréen , A. Krüger , & M. Spasojevic (Eds.), Pervasive computing (pp. 156–173). Springer. 10.1007/978-3-642-12654-3_10

[psyp14721-bib-0029] Hoemann, K. , Khan, Z. , Feldman, M. J. , Nielson, C. , Devlin, M. , Dy, J. , Barrett, L. F. , Wormwood, J. B. , & Quigley, K. S. (2020). Context‐aware experience sampling reveals the scale of variation in affective experience. Scientific Reports, 10, 12459. 10.1038/s41598-020-69180-y 32719368 PMC7385108

[psyp14721-bib-0030] Hoemann, K. , Wormwood, J. B. , Barrett, L. F. , & Quigley, K. S. (2023). Multimodal, idiographic ambulatory sensing will transform our understanding of emotion. Affective Science, 4(3), 480–486. 10.1007/s42761-023-00206-0 37744967 PMC10513989

[psyp14721-bib-0031] Houtveen, J. H. , & de Geus, E. J. C. (2009). Noninvasive psychophysiological ambulatory recordings: Study design and data analysis strategies. European Psychologist, 14(2), 132–141. 10.1027/1016-9040.14.2.132

[psyp14721-bib-0032] Ignatov, A. (2018). Real‐time human activity recognition from accelerometer data using convolutional neural networks. Applied Soft Computing, 62, 915–922. 10.1016/j.asoc.2017.09.027

[psyp14721-bib-0033] Jennings, J. R. , Kamarck, T. , Stewart, C. , Eddy, M. , & Johnson, P. (1992). Alternate cardiovascular baseline assessment techniques: Vanilla or resting baseline. Psychophysiology, 29(6), 742–750. 10.1111/j.1469-8986.1992.tb02052.x 1461961

[psyp14721-bib-0034] Kenney, M. J. , & Seals, D. R. (1993). Postexercise hypotension. Key features, mechanisms, and clinical significance. Hypertension (Dallas, Tex.: 1979), 22(5), 653–664. 10.1161/01.hyp.22.5.653 8225525

[psyp14721-bib-0035] Kivimäki, M. , & Steptoe, A. (2018). Effects of stress on the development and progression of cardiovascular disease. Nature Reviews. Cardiology, 15(4), 215–229. 10.1038/nrcardio.2017.189 29213140

[psyp14721-bib-0036] Klabunde, R. (2011). Cardiovascular physiology concepts. Lippincott Williams & Wilkins.

[psyp14721-bib-0037] Kusserow, M. , Amft, O. , & Tröster, G. (2013). Modeling arousal phases in daily living using wearable sensors. IEEE Transactions on Affective Computing, 4, 93–105. 10.1109/T-AFFC.2012.37

[psyp14721-bib-0038] Langer, A. W. , McCubbin, J. A. , Stoney, C. M. , Hutcheson, J. S. , Charlton, J. D. , & Obrist, P. A. (1985). Cardiopulmonary adjustments during exercise and an aversive reaction time task: Effects of Beta‐adrenoceptor blockade. Psychophysiology, 22(1), 59–68. 10.1111/j.1469-8986.1985.tb01561.x 3975320

[psyp14721-bib-0039] Linssen, L. , Landman, A. , van Baardewijk, J. U. , Bottenheft, C. , & Binsch, O. (2022). Using accelerometry and heart rate data for real‐time monitoring of soldiers' stress in a dynamic military virtual reality scenario. Multimedia Tools and Applications, 81(17), 24739–24756. 10.1007/s11042-022-12705-6

[psyp14721-bib-0040] Loeffler, S. , Hennig, J. , & Peper, M. (2016). Psychophysiological assessment of social stress in natural and laboratory situations: Using the experience sampling method and additional heart rate measures. Journal of Psychophysiology, 31, 1–11. 10.1027/0269-8803/a000170

[psyp14721-bib-0041] McEwen, B. S. (2006). Protective and damaging effects of stress mediators: Central role of the brain. Dialogues in Clinical Neuroscience, 8(4), 367–381. 17290796 10.31887/DCNS.2006.8.4/bmcewen PMC3181832

[psyp14721-bib-0042] Myrtek, M. (2004). Heart and emotion: Ambulatory monitoring studies in everyday life.

[psyp14721-bib-0043] Myrtek, M. , Aschenbrenner, E. , & Brügner, G. (2005). Emotions in everyday life: An ambulatory monitoring study with female students. Biological Psychology, 68(3), 237–255. 10.1016/j.biopsycho.2004.06.001 15620793

[psyp14721-bib-0044] Myrtek, M. , Brügner, G. , & Müller, W. (1996). Validation studies of emotional, mental, and physical workload components in the field. In Ambulatory assessment: Computer‐assisted psychological and psychophysiological methods in monitoring and field studies (pp. 287–304). Hogrefe & Huber Publishers.

[psyp14721-bib-0045] Myrtek, M. , & Spital, S. (1986). Psychophysiological response patterns to single, double, and triple stressors. Psychophysiology, 23(6), 663–671. 10.1111/j.1469-8986.1986.tb00690.x 3823342

[psyp14721-bib-0046] Nahum‐Shani, I. , Smith, S. N. , Spring, B. J. , Collins, L. M. , Witkiewitz, K. , Tewari, A. , & Murphy, S. A. (2017). Just‐in‐time adaptive interventions (JITAIs) in Mobile health: Key components and design principles for ongoing health behavior support. Annals of Behavioral Medicine: A Publication of the Society of Behavioral Medicine, 52(6), 446–462. 10.1007/s12160-016-9830-8 PMC5364076 27663578

[psyp14721-bib-0047] Nystoriak, M. A. , & Bhatnagar, A. (2018). Cardiovascular effects and benefits of exercise. Frontiers in Cardiovascular Medicine, 5, 135. 10.3389/fcvm.2018.00135 30324108 PMC6172294

[psyp14721-bib-0048] Obrist, P. A. (1976). The cardiovascular‐behavioral interaction—As it appears today. Psychophysiology, 13(2), 95–107. 10.1111/j.1469-8986.1976.tb00081.x 769018

[psyp14721-bib-0049] Patel, K. , Rössler, A. , Lackner, H. K. , Trozic, I. , Laing, C. , Lorr, D. , Green, D. A. , Hinghofer‐Szalkay, H. , & Goswami, N. (2016). Effect of postural changes on cardiovascular parameters across gender. Medicine, 95(28), e4149. 10.1097/MD.0000000000004149 27428203 PMC4956797

[psyp14721-bib-0050] Payne, R. L. , & Rick, J. T. (1986). Heart rate as an indicator of stress in suegeons and anaesthetists. Journal of Psychosomatic Research, 30(4), 411–420. 10.1016/0022-3999(86)90080-2 3761226

[psyp14721-bib-0051] Peçanha, T. , Bartels, R. , Brito, L. C. , Paula‐Ribeiro, M. , Oliveira, R. S. , & Goldberger, J. J. (2017). Methods of assessment of the post‐exercise cardiac autonomic recovery: A methodological review. International Journal of Cardiology, 227, 795–802. 10.1016/j.ijcard.2016.10.057 27836300

[psyp14721-bib-0052] Pedregosa, F. , Varoquaux, G. , Gramfort, A. , Michel, V. , Thirion, B. , Grisel, O. , Blondel, M. , Prettenhofer, P. , Weiss, R. , Dubourg, V. , Vanderplas, J. , Passos, A. , & Cournapeau, D. (2011). Scikit‐learn: Machine learning in python. Journal of Machine Learning Research, 12(Oct), 2825–2830.

[psyp14721-bib-0053] Pfaltz, M. C. , Kolodyazhniy, V. , Blechert, J. , Margraf, J. , Grossman, P. , & Wilhelm, F. H. (2015). Metabolic decoupling in daily life in patients with panic disorder and agoraphobia. Journal of Psychiatric Research, 68, 377–383. 10.1016/j.jpsychires.2015.04.027 26028550

[psyp14721-bib-0054] Pieper, S. , Brosschot, J. F. , van der Leeden, R. , & Thayer, J. F. (2007). Cardiac effects of momentary assessed worry episodes and stressful events. Psychosomatic Medicine, 69(9), 901–909. 10.1097/PSY.0b013e31815a9230 17991822

[psyp14721-bib-0055] Prill, T. , & Fahrenberg, J. (2007). New methods in ambulatory blood pressure monitoring: Interactive monitoring and detection of posture and movement patterns. Behavior Research Methods, 39(3), 390–398. 10.3758/bf03193008 17958150

[psyp14721-bib-0056] Qasem, L. , Cardew, A. , Wilson, A. , Griffiths, I. , Halsey, L. G. , Shepard, E. L. C. , Gleiss, A. C. , & Wilson, R. (2012). Tri‐axial dynamic acceleration as a proxy for animal energy expenditure; should we Be summing values or calculating the vector? PLoS One, 7(2), e31187. 10.1371/journal.pone.0031187 22363576 PMC3281952

[psyp14721-bib-0057] Romero, S. A. , Minson, C. T. , & Halliwill, J. R. (2017). The cardiovascular system after exercise. Journal of Applied Physiology, 122(4), 925–932. 10.1152/japplphysiol.00802.2016 28153943 PMC5407206

[psyp14721-bib-0058] Rosengren, A. , Hawken, S. , Ounpuu, S. , Sliwa, K. , Zubaid, M. , Almahmeed, W. A. , Blackett, K. N. , Sitthi‐amorn, C. , Sato, H. , Yusuf, S. , & INTERHEART investigators . (2004). Association of psychosocial risk factors with risk of acute myocardial infarction in 11119 cases and 13648 controls from 52 countries (the INTERHEART study): Case‐control study. Lancet (London, England), 364(9438), 953–962. 10.1016/S0140-6736(04)17019-0 15364186

[psyp14721-bib-0059] Rossberg, F. , & Peňaz, J. (1988). Initial cardiovascular response on change of posture from squatting to standing. European Journal of Applied Physiology and Occupational Physiology, 57(1), 93–97. 10.1007/BF00691245 3342800

[psyp14721-bib-0060] Roth, D. L. , Bachtler, S. D. , & Fillingim, R. B. (1990). Acute emotional and cardiovascular effects of stressful mental work during aerobic exercise. Psychophysiology, 27(6), 694–701. 10.1111/j.1469-8986.1990.tb03196.x 2100355

[psyp14721-bib-0061] Schrack, J. A. , Zipunnikov, V. , Goldsmith, J. , Bandeen‐Roche, K. , Crainiceanu, C. M. , & Ferrucci, L. (2014). Estimating energy expenditure from heart rate in older adults: A case for calibration. PLoS One, 9(4), e93520. 10.1371/journal.pone.0093520 24787146 PMC4005766

[psyp14721-bib-0062] Schultchen, D. , Reichenberger, J. , Mittl, T. , Weh, T. R. M. , Smyth, J. M. , Blechert, J. , & Pollatos, O. (2019). Bidirectional relationship of stress and affect with physical activity and healthy eating. British Journal of Health Psychology, 24(2), 315–333. 10.1111/bjhp.12355 30672069 PMC6767465

[psyp14721-bib-0063] Schwerdtfeger, A. R. , & Rominger, C. (2021). Feelings from the heart: Developing HRV decrease‐trigger algorithms via multilevel hyperplane simulation to detect psychosocially meaningful episodes in everyday life. Psychophysiology, 58(11), e13914. 10.1111/psyp.13914 34357598 PMC9285549

[psyp14721-bib-0064] Seiler, S. , Haugen, O. , & Kuffel, E. (2007). Autonomic recovery after exercise in trained athletes: Intensity and duration effects. Medicine and Science in Sports and Exercise, 39(8), 1366–1373. 10.1249/mss.0b013e318060f17d 17762370

[psyp14721-bib-0065] Shepherd, J. T. (1987). Circulatory response to exercise in health. Circulation, 76(6 Pt 2), VI3‐10. 3315298

[psyp14721-bib-0066] Sherwood, A. , Allen, M. T. , Obrist, P. A. , & Langer, A. W. (1986). Evaluation of Beta‐adrenergic influences on cardiovascular and metabolic adjustments to physical and psychological stress. Psychophysiology, 23(1), 89–104. 10.1111/j.1469-8986.1986.tb00602.x 3003780

[psyp14721-bib-0067] Sims, J. , & Carroll, D. (1990). Cardiovascular and metabolic activity at rest and during psychological and physical challenge in normotensives and subjects with mildly elevated blood pressure. Psychophysiology, 27(2), 149–156. 10.1111/j.1469-8986.1990.tb00366.x 2247546

[psyp14721-bib-0068] Stults‐Kolehmainen, M. A. , & Sinha, R. (2014). The effects of stress on physical activity and exercise. Sports Medicine (Auckland, N.Z.), 44, 81–121. 10.1007/s40279-013-0090-5 24030837 PMC3894304

[psyp14721-bib-0069] Su, T. , Sun, H. , Ma, C. , Jiang, L. , & Xu, T. (2019). HDL: Hierarchical deep learning model based human activity recognition using smartphone sensors. International Joint Conference on Neural Networks (IJCNN), 2019, 1–8. 10.1109/IJCNN.2019.8851889

[psyp14721-bib-0070] Tufek, N. , Yalcin, M. , Altintas, M. , Kalaoglu, F. , Li, Y. , & Bahadir, S. K. (2020). Human action recognition using deep learning methods on limited sensory data. IEEE Sensors Journal, 20, 3101–3112. 10.1109/JSEN.2019.2956901

[psyp14721-bib-0071] Turner, J. R. , & Carroll, D. (1985). Heart rate and oxygen consumption during mental arithmetic, a video game, and graded exercise: Further evidence of metabolically‐exaggerated cardiac adjustments? Psychophysiology, 22(3), 261–267. 10.1111/j.1469-8986.1985.tb01597.x 4011795

[psyp14721-bib-0072] Turner, J. R. , Carroll, D. , & Courtney, H. (1983). Cardiac and metabolic responses to “space invaders”: An instance of metabolically‐exaggerated cardiac adjustment? Psychophysiology, 20(5), 544–549. 10.1111/j.1469-8986.1983.tb03010.x 6635093

[psyp14721-bib-0073] Turner, J. R. , Carroll, D. , Hanson, J. , & Sims, J. (1988). A comparison of additional heart rates during active psychological challenge calculated from upper body and lower body dynamic exercise. Psychophysiology, 25(2), 209–216. 10.1111/j.1469-8986.1988.tb00990.x 3399609

[psyp14721-bib-0074] van der Mee, D. J. , Gevonden, M. J. , Westerink, J. H. D. M. , & de Geus, E. J. C. (2021). Validity of electrodermal activity‐based measures of sympathetic nervous system activity from a wrist‐worn device. International Journal of Psychophysiology, 168, 52–64. 10.1016/j.ijpsycho.2021.08.003 34418464

[psyp14721-bib-0075] Vella, C. , & Robergs, R. (2005). A review of the stroke volume response to upright exercise in healthy subjects. British Journal of Sports Medicine, 39(4), 190–195. 10.1136/bjsm.2004.013037 15793084 PMC1725174

[psyp14721-bib-0076] Verkuil, B. , Brosschot, J. F. , Tollenaar, M. S. , Lane, R. D. , & Thayer, J. F. (2016). Prolonged non‐metabolic heart rate variability reduction as a physiological marker of psychological stress in daily life. Annals of Behavioral Medicine, 50(5), 704–714. 10.1007/s12160-016-9795-7 27150960 PMC5054058

[psyp14721-bib-0077] Vrijkotte, T. G. , van Doornen, L. J. , & de Geus, E. J. (2000). Effects of work stress on ambulatory blood pressure, heart rate, and heart rate variability. Hypertension (Dallas, Tex.: 1979), 35(4), 880–886. 10.1161/01.hyp.35.4.880 10775555

[psyp14721-bib-0078] Wang, K. , He, J. , & Zhang, L. (2019). Attention‐based convolutional neural network for weakly labeled human activities recognition with wearable sensors. IEEE Sensors Journal, 19(17), 7598–7604. 10.1109/JSEN.2019.2917225

[psyp14721-bib-0079] Wilhelm, F. H. , & Roth, W. T. (1998). Using minute ventilation for ambulatory estimation of additional heart rate. Biological Psychology, 49(1), 137–150. 10.1016/S0301-0511(98)00032-5 9792490

[psyp14721-bib-0080] Yang, C.‐C. , & Hsu, Y.‐L. (2010). A review of accelerometry‐based wearable motion detectors for physical activity monitoring. Sensors, 10(8), 7772–7788. 10.3390/s100807772 22163626 PMC3231187

[psyp14721-bib-0081] Yang, Z. , Jia, W. , Liu, G. , & Sun, M. (2017). Quantifying mental arousal levels in daily living using additional heart rate. Biomedical Signal Processing and Control, 33, 368–378. 10.1016/j.bspc.2016.11.003

[psyp14721-bib-0082] Zanstra, Y. J. , & Johnston, D. W. (2011). Cardiovascular reactivity in real life settings: Measurement, mechanisms and meaning. Biological Psychology, 86(2), 98–105. 10.1016/j.biopsycho.2010.05.002 20561941 PMC3131085

